# Heterologous constitutive production of short-chain-length polyhydroxyalkanoates in *Pseudomonas putida* KT2440: the involvement of IbpA inclusion body protein

**DOI:** 10.3389/fbioe.2023.1275036

**Published:** 2023-11-01

**Authors:** Maria-Tsampika Manoli, Francisco G. Blanco, Virginia Rivero-Buceta, Ryan Kniewel, Sandra Herrera Alarcon, Sergio Salgado, M. Auxiliadora Prieto

**Affiliations:** ^1^ Interdisciplinary Platform for Sustainable Plastics Towards a Circular Economy-Spanish National Research Council (SusPlast-CSIC), Madrid, Spain; ^2^ Polymer Biotechnology Group, Department of Microbial and Plant Biotechnology, Margarita Salas Center for Biological Research (CIB-CSIC), Madrid, Spain

**Keywords:** *Pseudomonas* putida, synthetic biology, polyhydroxyalkanoates, modular cloning assembly, IbpA inclusion body protein

## Abstract

Designing cell factories for the production of novel polyhydroxyalkanoates (PHAs) via smart metabolic engineering is key to obtain *à la carte* materials with tailored physicochemical properties. To this end, we used the model medium-chain-length-PHA producing bacterium, *P. putida* KT2440 as a chassis, which is characterized by its metabolic versatility and stress tolerance. Different PHA biosynthetic modules were assembled in expression plasmids using the Golden gate/MoClo modular assembly technique to implement an orthogonal short-chain-lengh-PHA (scl-PHA) switch in a “deaf” PHA mutant. This was specifically constructed to override endogenous multilevel regulation of PHA synthesis in the native strain. We generated a panel of engineered approaches carrying the genes from *Rhodospirillum rubrum, Cupriavidus necator* and *Pseudomonas pseudoalcaligenes,* demonstrating that diverse scl-PHAs can be constitutively produced in the chassis strain to varying yields from 23% to 84% PHA/CDW. Co-feeding assays of the most promising engineered strain harboring the PHA machinery from *C. necator* resulted to a panel of PHBV from 0.6% to 19% C5 monomeric incorporation. Chromosomally integrated PHA machineries with high PhaC^Cn^ synthase dosage successfully resulted in 68% PHA/CDW production. Interestingly, an inverse relationship between PhaC synthase dosage and granule size distribution was demonstrated in the heterologous host. In this vein, it is proposed the key involvement of inclusion body protein IbpA to the heterologous production of tailored PHA in *P. putida* KT2440.

## 1 Introduction

**FIGURE 1:**
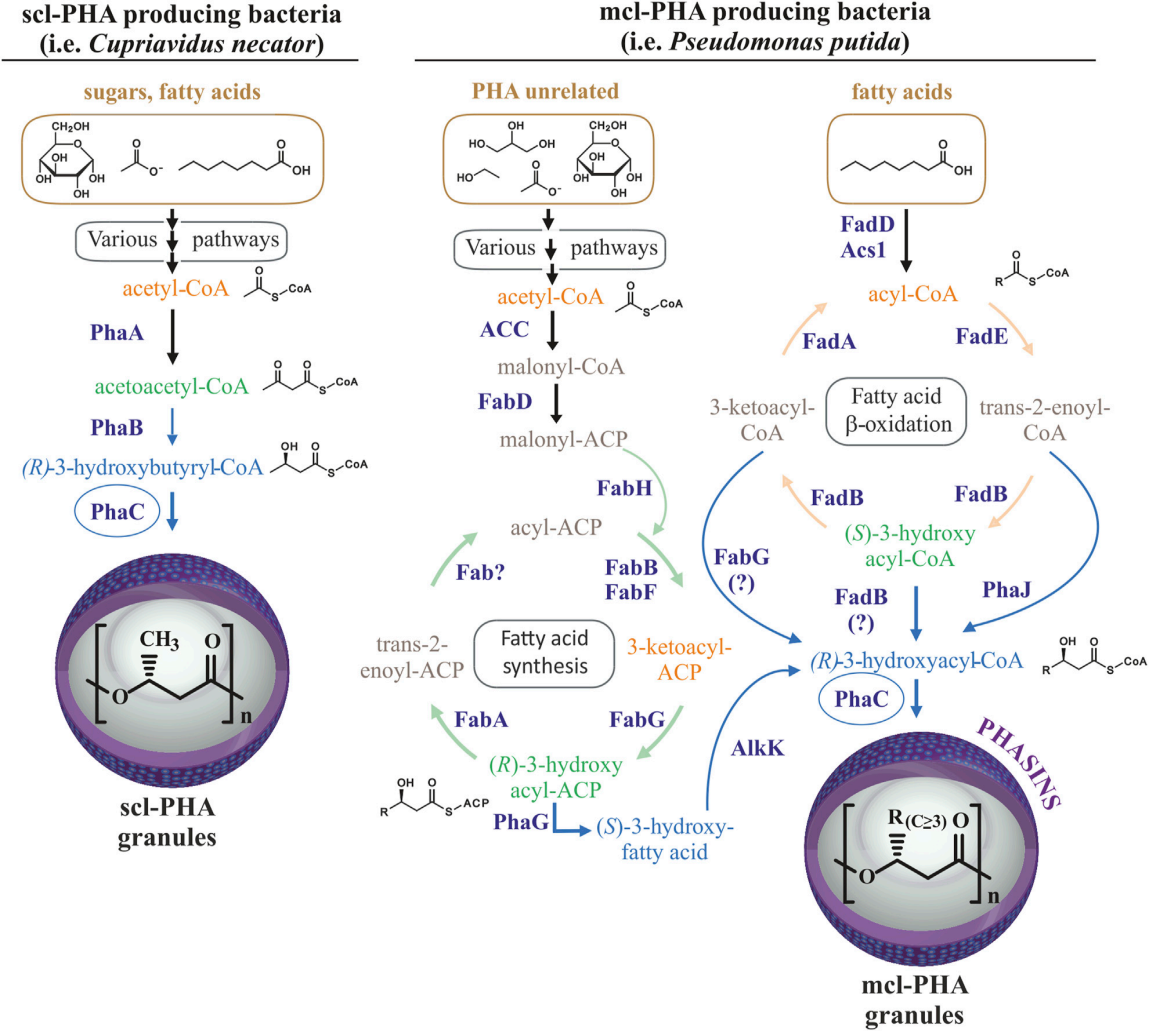
Routes for PHA production. Comparison of routes for scl-PHA production in many bacteria (left) and mcl-PHA production in *Pseudomonas putida* (right). Scl-PHA production requires the PhaA 3-ketothiolase and PhaB acetoacetyl-CoA reductase in addition to the PhaC synthase. Mcl-PHA 3-hydroxyacyl monomers are produced in *Pseudomonas putida* by *de novo* synthesis of fatty acids from PHA-unrelated substrates and *ß*-oxidation of fatty acids, allowing for the production of mcl-PHA from diverse carbon sources from diverse carbon sources (shown with brownish color).

Polyhydroxyalkanoates (PHAs) are biotechnologically useful natural polyesters produced in many microorganisms. PHAs function as an intracellular carbon and energy storage reservoir with physical and mechanical properties that make them promising bioplastics with many possible applications (Zheng et al., 2019). Optimal PHA production generally occurs when a deficit exists in the nutritional conditions of the cell, with the deprivation of nitrogen being the most widely applied in bioproduction strategies (Kim and Lenz, 2001; Mahato et al., 2021; Ronďošová et al., 2022). Hydrophobic PHA accumulates in roughly spherical cellular inclusions called PHA granules that are segregated from the cytoplasm by a surface layer of amphipathic phasin proteins and other granule-associated proteins (Jendrossek, 2009). The Gram-negative *P. putida* KT2440 has been extensively studied as archetypal producer of medium-chain-length PHA (mcl-PHA) containing monomers of 6–14 carbon atoms in length (C6-C14) (Mezzina et al., 2021). The *P. putida pha* genomic locus contains the genetic machinery (Supplementary Figure S1, panel A) which along with its metabolic flexibility, allows for the production of mcl-PHA from a variety of substrates, including both PHA-like aliphatic fatty acids as well as PHA-unrelated carbon sources (Figure 1) (Cheng and Charles, 2016; Koller et al., 2017).

In contrast to *P. putida*, many bacterial species are capable of producing short-chain-length PHA (scl-PHA) with monomers of 3–5 carbon atoms in length (C3-C5) from PHA-unrelated carbon sources (Figure 1). For example, the production of polyhydroxybutyrate (PHB) from sugars in *C. necator* requires the 3-ketothiolase PhaA to condense two glycolysis-derived acetyl-CoA molecules into acetoacetyl-CoA, and the acetoacetyl-CoA reductase PhaB to reduce acetoacetyl-CoA to four carbon 3-hydroxybutyryl-CoA, the substrate for PhaC synthase (Figure 1). Acetyl-CoA for PHB synthesis can also be provided by other carbon sources, such as the degradation of amino acids or *ß*-oxidation of fatty acids (Jendrossek and Pfeiffer, 2014). Some bacteria, such as *Rhodospirillum rubrum*, are capable of producing heteropolymers of both, scl-PHA with monomers of 4 and 5 carbon atoms, as well as mcl-PHA by incorporating 3-hydroxyhexanoate-CoA or 3-hydroxyheptanoate-CoA monomers when provided with medium-chain-length fatty acids (hexanoate) as the carbon source (Brandl et al., 1989; Jin and Nikolau, 2012; Godoy et al., 2023). Importantly, in addition to the supplied substrate and metabolic determinants, organism-specific PhaC synthase(s) determine the length monomers that can be incorporated into the polymer due to differences in substrate specificity (Kim et al., 2017). Thus, the class I PHA synthases, PhaC1 from *Cupriavidus necator* and PhaC2 from *R. rubrum* are generally able to incorporate monomers containing up to 5 or 7 carbons in length, respectively (Brandl et al., 1989). In contrast, the class II PHA synthases from *P. putida* can incorporate a wide variety of monomers of lengths between 6 and 14 carbons to produce mcl-PHAs. As mentioned above, wild type *P. putida* does not naturally produce scl-PHAs, which is likely due to the substrate specificity of its PHA synthases along with a metabolic propensity to produce longer chain 3-hydroxyacyl-CoA monomers (Huisman et al., 1989; Kim et al., 2017).

Pseudomonads gained special interest due to their metabolic versatility, adaptability to endogenous and exogenous stresses. Specifically, *P. putida* KT2440 has become a model organism for biotechnological, environmental and industrial applications due to the presence of different genome-scale metabolic models and high advances in synthetic biology and metabolic engineering fields (Mezzina et al., 2021). In fact, several studies have demonstrated the use of *P. putida* as a chassis for the heterologous expression of scl- or mcl-PHA machinery from other bacteria (Matsusaki et al., 1998; Clemente et al., 2000; Ouyang et al., 2007; Cha et al., 2020). For orthogonal scl-PHA production in this strain apart from the deletion of the native *pha* locus, the expression of *phb* genes is required (Figure 1) (Prieto et al., 2016). However, the majority of these studies were based on the heterologous inducible expression systems of *phb* gene clusters, which are not useful for scaling up processes. Digging into the optimization of the constitutive scl-PHA production in *P. putida,* in this work we have used as microbial chassis the PP05_01 strain with the entire native *pha* genomic locus deleted (Mato et al., 2020). To enhance the capacity for designing, building and testing of heterologous constitutive expression systems with different strengths, we adapted our Golden gate/MoClo modular assembly cloning method for obtaining plasmids suited for constitutive PHA production at different rates in *P. putida* (Blázquez et al., 2023). These approaches allowed us not only to control the monomer composition, but to identify the inclusion body protein IbpA as an important partner for heterologous production of scl-PHA in *P. putida* KT2440. We demonstrated an inverse relationship between PhaC synthase dosage and granule size/number distribution in the heterologous host driven by IbpA.

## 2 Materials and methods

### 2.1 Bacterial strains, media and culture conditions

Bacterial strains and plasmids used in this work are listed in Table 1 and Supplementary Table S1 respectively. Unless otherwise indicated, *E. coli* and *P. putida* strains pre-cultures were grown in lysogeny broth (LB) at 37°C and 30°C respectively, at 200 rpm. Streptomycin (75 μg/mL), ampicillin (100 μg/mL), kanamycin (50 μg/mL), gentamycin (10 μg/mL), chloramphenicol (34 μg/mL), IPTG (0.5–1 mM) and Xgal (40 μg/mL) were added as required. Solid media were made by the addition of 1.5% (w/v) agar.

**TABLE 1:** Strains used in this study.

Strains	Relevant characteristics	References
*Pseudomonas putida*		
KT2440	Wild-type strain derived from *P. putida* mt-2 cured of the pWW0 plasmid	Bagdasarian et al. (1981)
PP05_01	KT2440 derivative strain with *pha* cluster deleted (PP_5003-PP_5008) _CECT 30020	Mato et al. (2020)
PP00_02	Km^r^, PP05_01 derivative strain expressing P*trc*:*phaC* ^*Cn*^ *-phaA* ^*Cn*^ *-phaB* ^*Cn*^ genes, integrated into the genome (via mini-Tn*5* transposon) using the pMAB26 plasmid	This work
PP00_03	Km^r^, KT2440 strain expressing P*trc*:*phaC* ^*Cn*^ *-phaA* ^*Cn*^ *-phaB* ^*Cn*^ genes integrated into the genome (via mini-Tn*5* transposon) using the pMAB26 plasmid	This work
PP01_02	Gm^r^, PP05_01 derivative strain harboring 14f:BCD2-*phaC* ^*Cn*^-*rnpB*T1; 14a:BCD2-*phaA* ^*Cn*^-*rpoC*;14a:BCD2-*phaB* ^*Cn*^-λT1 at *att*Tn*7* site	This work
PP05_12	Gm^r^, PP05_01 derivative strain harboring 14a:BCD2-*phaC* ^*Cn*^-*rnpB*T1; 14a:BCD2-*phaA* ^*Cn*^-*rpoC*;14a:BCD2-*phaB* ^*Cn*^-λT1, at *att*Tn*7* site	This work
PP05_15	Gm^r^, PP01_02 derivative strain with *ibpA* locus deleted (PP_1982)	This work
PP05_16	Gm^r^, PP05_01 derivative strain harboring 14a:BCD2-*phaC* ^*Cn*^-*rnpB*T1; 14a:BCD2-*phaA* ^*Cn*^-*rpoC*;14a:BCD2-*phaB* ^*Cn*^-λT1; 14a:BCD2-*phaP1* ^*Cn*^-*rnpB*T1, at *att*Tn*7* site	This work
KT2440 (pGG128)	Km^r^, KT2440 strain harboring pGG128 empty plasmid	This work
PP05_01 (pGG128)	Km^r^, PP05_01 strain harboring pGG128 empty plasmid	This work
PP05_01 (pSS126)	Km^r^, PP05_01 strain harboring pSS126; 14a:BCD2-*phaC2* ^*Rr*^-λT0; 14a:BCD2-*phaA* ^*Rr*^-λT0; 14a:BCD2-*phaB* ^*Rr*^- λT0	This work
PP05_01 (pRK216)	Km^r^, PP05_01 strain harboring pRK216; SynPro16-*phaC* ^*Cn*^-*rnpB*T1; SynPro16-*phaA* ^*Cn*^-*rpoC*; SynPro16-*phaB1* ^*Cn*^-T500	This work
PP05_01 (pMM85)	Km^r^, PP05_01 strain harboring pMM85; 14a:BCD2-*phaC* ^*Cn*^ *-rnpB*T1; 14a:BCD2-*phaA* ^*Cn*^-*rpoC*; 14a:BCD2-*phaB* ^*Cn*^ *-*λT1; 14a:BCD2-*phaP1* ^*Cn*^-*rnpB*T1	This work
PP05_01 (pMM106)	Km^r^, PP05_01 strain harboring pMM106; 14a:BCD2-*phaC5* ^*Pp*^ *-rnpB*T1; 14a:BCD2-*phaA3* ^*Pp*^-*rpoC*; 14a:BCD2-*phaB* ^*Pp*^-λT1; 14a:BCD2-*phaP1* ^*Pp*^-*rnpB*T1	This work
PP05_12 (pMM194)	Km^r^, Gm^r^, PP05_12 strain harboring pMM194; 14a:BCD2-*ibpA* ^*Pp*^-*rnpB*T1	This work
PP05_15 (pMM194)	Km^r^, Gm^r^, PP05_15 strain harboring pMM194; 14a:BCD2-*ibpA* ^*Pp*^-*rnpB*T1	This work
PP01_02 (pBDN2-GFP)	Km^r^, PP01_02 strain harboring pBDN2-GFP, empty plasmid	Blanco et al. (2023)
PP01_02 (pBDN2-PhaP)	Km^r^, PP01_02 strain harboring pBDN2-*PhaP*, harboring the *PhaP1* ^*Cn*^	Blanco et al. (2023)
*Escherichia coli*		
DH5αλ*pir*	Tc^r^, cloning host; DH5α lysogenized with λ*pir* phage. Host strain for *ori*R6K plasmids	Zobel et al. (2015)
DH10B	Sp^r^, cloning host; F-, *mcrA* Δ(*mrr hsdRMS-mcrBC*) Φ80d*lac*ΔM15 Δ*lacX74 deoR recA1 araD139* Δ(*ara-leu*)7697	Invitrogen, Thermo Fisher Scientific, United States of America
HB101 (pRK600)	Cm^r^, Conjugation helper strain; F^−^ λ^−^ *hsdS20*(*r*_*B*_^*-*^ *m*_*B*_^*-*^) *recA13 leuB6*(Am) *araC14* Δ(*gpt-proA*)62 *lacY1 galK2*(Oc) *xyl-5 mtl-1 rpsL20*(Sm^r^) *glnX44* (AS)	Boyer and Roulland-Dussoix (1969)
*R. rubrum* ATCC 11170	*R. rubrum* type strain	Pfennig and Trüper (1971)
*C. necator* H16 DSM 428	*C. necator* H16	Makkar and Casida (1987)

For PHA production standard laboratory methods were performed as previously described (Manoli et al., 2022). Briefly, *P. putida* strains were grown overnight in LB, the cells were washed twice with 0.85% saline solution and adjusted to an optical density of 600 nm of 0.3. Then, *P. putida* cells were grown for 24 h at 30°C and 200 rpm in 0.1 N M63, a nitrogen-limited minimal medium (13.6 g/L KH_2_PO_4_, 0.2 g/L (NH_4_)_2_SO_4_, 0.5 mg/L FeSO_4_·7H_2_O, adjusted to pH 7.0 with KOH). This medium was supplemented with 1 mM MgSO_4_, a solution of trace elements/Goodies (composition 1000X: 2.78 g FeSO_4_·7H_2_O, 1.98 g MnCl_2_·4H_2_O, 2.81 g CoSO_4_·7H_2_O, 1.47 g CaCl_2_·2H_2_O, 0.17 g CuCl_2_·2H_2_O, 0.29 g ZnSO_4_·7H_2_O dissolved in 1 L water), 15 mM sodium octanoate or 20 mM glucose as the carbon source (C/N ratio was maintained at 40 mol/mol). Concerning the inducible P*trc* cultures 1 mM IPTG was used from the beginning of the assays. Culture growth was monitored in shaking Erlenmeyer flasks of 250 mL (maintaining a volume to air ratio of 1/5) by measuring optical density at 600 nm (OD600) using a portable spectrophotometer (ThermoFisher Scientific).

### 2.2 Transformation of *Pseudomonas putida* strains by electroporation

*Pseudomonas putida* strains were transformed following the protocol described by Choi et al. with some adaptations (Choi et al., 2006). Briefly, the strains of *P. putida* KT2440 and PP05_01 were grown overnight in 5 mL of LB at 30°C and 200 rpm and subcultured into 50 mL of LB to an OD600 between 0.5–1.0. These cultures were pelleted at 3,200 ×*g* for 8 min, washed five times with 300 mM sucrose and resuspended in 500 μL of 300 mM sucrose. 100 μL of cell suspension were mixed with 100 ng of desired plasmid and transferred to a 2 mm gap electroporation cuvette. After a pulse of 25 μF, 2.5 kV and 200 Ω; 900 μL of room temperature LB was added and transferred to a 100 × 16 mm round-bottom polypropylene tube and incubated for 1 h at 30°C, 200 rpm. 5 μL and 100 μL of the transformation cultures were plated on LB agar plates containing the corresponding antibiotic for the plasmid maintenance and grown at 30°C.

### 2.3 Molecular biology reagents

Plasmid DNA minipreps were made using the High Pure Plasmid Isolation Kit (Roche) following the manufacturer’s protocol. Genomic extractions of *P. putida* KT2440, *R. rubrum* ATCC11170, *C. necator* H16 and *P*. *pseudoalcaligenes* CECT5344 were performed with the illustra bacteria genomicPrep Mini Spin Kit (GE Healthcare). DNA agarose gel bands, PCR products and digestion products were purified with illustraTM GFX PCR DNA and Gel Band Purification Kit (GE Healthcare). DNA concentration was measured using a NanoDrop 2000 Spectrophotometer (ThermoFisher Scientific). The Golden gate restriction enzymes, BbsI (BpiI) and BsaI (Eco31I) were from ThermoFisher Scientific. Phusion DNA polymerase, T4 DNA ligase and all other restriction enzymes were from New England Biolabs. The MoClo toolkit was a gift from Sylvestre Marillonnet via Addgene (Kit #1000000044) (Weber et al., 2011; Werner et al., 2012).

### 2.4 Adapted golden gate/MoClo protocol

**FIGURE 2:**
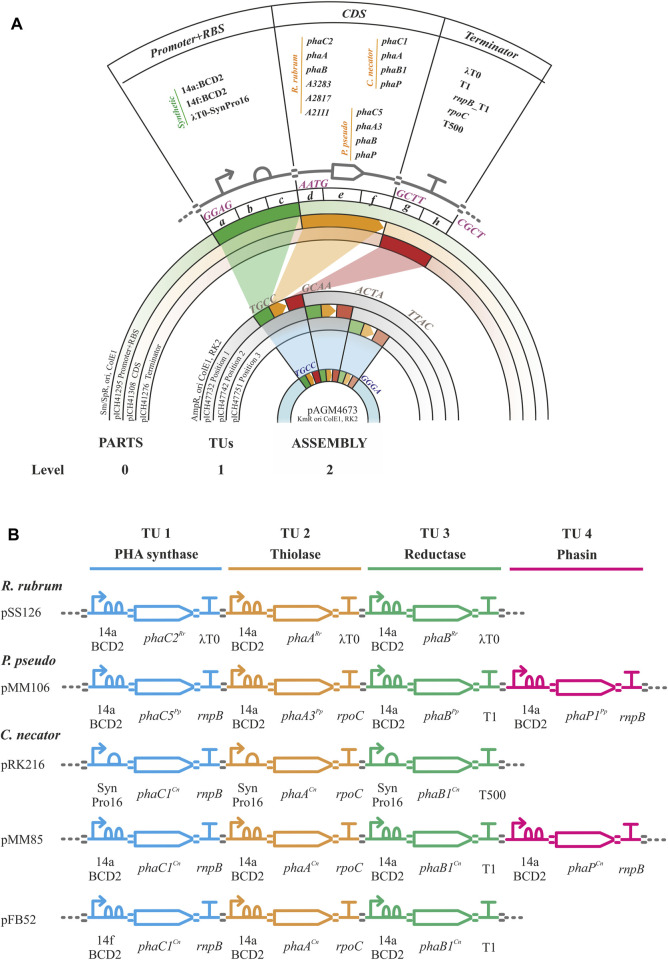
Golden gate/MoClo schema. **(A)**. Golden gate/MoClo assembly cloning schema of levels 0, 1 and 2 as visualized by concentric plasmid constructs for the creation of a typical assembly containing three transcription units (TUs). Level 0: Promoter, CDS, and terminator parts cloned into level 0 plasmids pICH41295, pICH41308 and pICH41276, respectively. The Level 0 parts used are shown. The four nucleotide 5′ overhang BbsI ligation fusion sites are indicated for each level 0 part. Level 1: Three TU constructs in level 1 plasmids for positions 1, 2 and 3 by plasmids pICH4732, pICH47741 and pICH47751, respectively. Four nucleotide 5′ overhang BsaI ligation fusion sites for linking each of the TUs are indicated. Level 2: Final assembly of three TUs into plasmid pAGM4673 along with the inclusion of the end-linker from plasmid pICH41780 (not shown, provides fusion sites for ligation of 5′ TTAC and 5′ GGGA) to occupy positions 4–7. Four nucleotide 5′ overhang BbsI ligation fusion sites flanking the three TUs are indicated. **(B)**. Example genetic design schematics of Golden gate/MoClo level 2 plasmid assemblies for the expression of PHB machinery from *R. rubrum* (pSS126), *Pseudomonas pseudoalcaligenes* (pMM106) and *Cupriavidus necator* (pRK216, pMM85, pFB52). The TU assemblies contain the PHA synthase, thiolase, reductase, and optional phasin genes with their promoters and terminators indicated. Genetic design glyphs follow the Synthetic Biology Open Language (SBOL) visual standard for elements: promoter, RBS, CDS, terminator and assembly scar.

Golden gate/MoClo assembly is a hierarchical method requiring the establishment of a library of promoter + RBS, CDS and terminator parts (level 0) that are assembled into higher order promoter-CDS-terminator transcription units (TU, level 1) and finally into assemblies containing 1 to 7 transcription units (level 2, Figure 2). The parts comprised the essential units needed to assemble the heterologous expression constructs for PHA production (i.e., *phaC* synthase, *phaA* 3-ketothiolase, *phaB* acetoacetyl-CoA, *phaP* phasin) from *C. necator, P. pseudoalcaligenes* and *R. rubrum.* Non-CDS parts included synthetic constitutive promoters of varying strengths. These promoters contained RBS sequences by the inclusion of the two Shine-Dalgarno sequences/RBSs in the bicistronic translational coupler BCD2 for the low expression promoter BG28/14a from Zobel *et al.* (level 0 plasmid pSS15) or the AGGGGG RBS for the moderate expression promoter SynPro16 from Tiso *et al.* (level 0 plasmid pRK154) (Zobel et al., 2015; Tiso et al., 2016). Following the design of SynPro16 validated by Tiso et al., the presence of the upstream insulating terminator λT0 was maintained in this promoter part. These two promoters were chosen for the assemblies used in the proof-of-concept experiments described below as they were expected to confer low and moderate constitutive expression in *P. putida* due to the published descriptions of their activities (Zobel et al., 2015; Tiso et al., 2016). Finally, parts were made for five rho-independent terminators with efficiencies of ≥98%, including the natural terminators λT0, *rrnB*_T1, *rnpB*_T1, *rpoC* and the synthetic T500 (Stueber and Bujard, 1982; Lutz and Bujard, 1997; Yarnell and Roberts, 1999; Larson et al., 2008; Cambray et al., 2013).

Golden gate/MoClo plasmids were constructed following Weber et al. with some modifications and recently updated by Weber et al. (2011), Blázquez et al. (2023). For Level 0 plasmid construction, every part was PCR amplified with DNA oligos designed using Benchling (www.benchling.com) with the following characteristics: a tail containing the BbsI recognition site followed by the corresponding four nucleotide fusion site, 21 bp of minimal length for target complementarity, 50°C of minimal T_m_ for that region and a maximal T_m_ difference of ±1.5°C between both oligos. Promoter and CDS sequences were PCR amplified from *C. necator* H16, *R. rubrum* ATCC11170, *P. pseudoalcaligenes* CECT5344 and *P. putida* KT2440 genomic DNA or plasmid templates. Terminator sequences were PCR amplified from *E. coli* DH5α genomic DNA or plasmid templates. Oligo primers, templates and references for all Golden gate level 0 PCR amplifications are listed in Supplementary Table S2. Where needed DNA domestication (removal of naturally present internal BbsI or BsaI restriction sites was carried out using the strategy of Engler et al. (2008). This strategy generated PCR subparts with 5’ cohesive end fusion sites overlapping the internal restriction sites to be eliminated. The primers used for PCR contained synonymous substitution point mutations in the fusion nucleotides of a BbsI site that eliminate the restriction site upon ligation in the golden gate level 0 reaction. PCR products were purified using a gel purification kit following manufacturer instructions. Golden gate digestion-ligation reactions were set up with 100 ng of level 0 acceptor plasmid (position *abc*, pICH41295; position *def*, pICH41308; or position *gh*, pICH41276; Supplementary Table S1) and the corresponding amount of gel purified PCR product to achieve a 2:1 insert-vector molar ratio; 10 U BbsI, 400 U T4 DNA ligase and 1 mM ATP in Buffer G (10 mM Tris-HCl pH 7.5, 10 mM MgCl_2_, 50 mM NaCl and 0.1 mg/mL bovine serum albumin; ThermoFisher Scientific) in a reaction volume of 20 μL. The reaction was incubated in a thermocycler with four cycles of 37°C for 10 min and 16°C for 10 min followed by 65°C for 20 min. A 100 μL aliquot of chemically competent *E. coli* DH5α was then transformed by heat shock with 5 μL of the Golden gate reaction. Transformed cells were plated on LB-agar supplementedwith 75 μg/mL streptomycin, 0.5 mM IPTG and 40 μL/mL X-gal and grown overnight at 37°C for selection of the disruption of *a*-complementation *ß*-galactosidase activity. Several white colonies per transformation were transferred to 4 mL LB medium with 75 μg/mL streptomycin and grown overnight at 37°C for plasmid purification. The extracted plasmids were digested with BsaI to check for the presence of the correct insert size and confirmed by sequencing with primers RK81 and RK82 (Supplementary Table S2).

For Level 1 construction of TUs, the reaction mix contained 100 ng of acceptor plasmid (depending on the TU position, Supplementary Table S1), the three level 0 plasmids containing a promoter plasmid part, a CDS plasmid part and a terminator plasmid part in a 2:1 donor plasmid:acceptor plasmid molar ratio; 10 U BsaI, 400 U T4 DNA ligase and 1 mM ATP in Buffer G in a reaction volume of 20 μL. The reaction was incubated in a thermocycler for four cycles of 40°C for 10 min and 16°C for 10 min, then at 50°C for 10 min and 80°C for 20 min. Reactions were transformed into *E. coli* DH5α and plasmids prepared as for level 0 golden gate reactions except that 100 μg/mL ampicillin, 0.5 mM IPTG and 40 μL/mL X-gal were present for selection. The extracted plasmids were digested with BbsI to check for the presence of the correct insert size and sequenced with primers RK155 and RK156 (Supplementary Table S2).

Level 2 assembly reactions were carried out with 100 ng of acceptor plasmid (pAGM4673, RK2 origin of replication providing some range in copy number in pseudomonads estimated to be maintained at 30 ± 10 copies per genome equivalent in *P. putida*, Supplementary Table S1), the corresponding end-linker plasmid depending on the number of TUs to be inserted, each level 1 plasmid in a 2:1 donor plasmid:acceptor plasmid molar ratio, 10 U BbsI, 400 U T4 DNA ligase and 1 mM ATP in Buffer G in a reaction volume of 20 μL. The reaction was incubated in a thermocycler with four cycles of 37°C for 10 min and 16°C for 10 min followed by 65°C for 20 min. Reactions were transformed into *E. coli* DH5α as for level 0 golden gate reactions except that 50 μg/mL kanamycin was added for selection. Red-white color selection was carried out (red color canthaxanthin produced by its operon in the cloning site of pAGM4673) and several white colonies were transferred to 4 mL of liquid LB medium with 50 μg/mL kanamycin for plasmid minipreps. The extracted plasmids were confirmed by digestion with DraIII or EcoRI and sequenced using primers RK157 and RK158 (Supplementary Table S2).

### 2.5 Strains construction for deletion, complementation assays, and chromosomic integration

For *ibpA* gene deletion, the pEMG knockout system was used, generating the PP05_15 strain. *ibpA* encodes a small heat shock protein, located in PP_1982 locus. Two pairs of primers (MM388-MM389, MM390-MM391, Supplementary Table S2) were designed to amplify the flanking fragments of the *ibpA* locus. An overlap PCR was carried out giving a product of 1.6 kb and was cloned into pEMG plasmid using EcoRI and BamHI restriction enzymes. For the first and second recombination steps, we followed the protocol described above. In this case for the confirmation of the second recombination event, external pair of primers (i.e., MM392-MM393) of to be deleted region were used.

For IbpA complementation assays, the PP05_15 strain was transformed with pMM194 plasmid (obtained using Golden gate/MoClo strategy) that contains the *ibpA* gene under the low strength 14a promoter and the BCD2 element. PHA accumulation assays were then performed as stated below.

For the co-localization experiments one multipurpose vector was constructed, with a phasin fusion position tags and under the control of the inducible *Pm* promoter. For the *in vivo* co-localization experiments the pBDN2 vector was used, since it contains the cloning site for the phasin between two SapI restriction sites (Blanco et al., 2023). Then, PP01_02 strain was transformed with the pBDN2 derived plasmids (i.e., empty, including only the msf-GFP, as negative control and harboring the wild type *PhaP1* ^*Cn*^). The resulting strains were inoculated at OD600 nm of 0.3 under PHA accumulating conditions and were allowed to grow until the polymer accumulation was visible (i.e., OD600 = 0.7). At this point the cultures were induced with 1 mM 3 MB for the expression of the fusion protein phasin-GFP (Mato et al., 2020).

To facilitate the single-copy genes into bacterial chromosome (i.e., for the generation of PP05_12 and PP01_02 strains), an adapted for Golden gate broad host range mini-Tn*7* vector (pRK99) was used. The genome integration is based on a neutral and naturally evolved *att*Tn*7* site, located downstream of a highly conserved *glmS* gene (Zobel et al., 2015). A four parental mating process was carried out from overnight LB precultures of *E. coli* CC118λpir bearing pMM175 and pFB52 plasmids (donor strains), *E. coli* HB101 (pRK600) (helper strain), *E. coli* DH5aλpir (pTnS-1) (leading transposase strain), and *P. putida* PP05_01 (recipient strain). The transconjugants were selected on cetrimide agar containing 10 μg/mL gentamycin plates and incubated at 30°C for 18 h. The next day few colonies were picked on LB kanamycin, to verify the loss of the plasmid and LB gentamycin and incubated at 30°C for 18 h. Few gentamycin resistant and kanamycin sensitive clones were selected to verify the correct insertion of the transposon into the *att*Tn*7* stie and checked via colony PCR and sequencing (Zobel et al., 2015).

### 2.6 PHA quantification and monomer composition

For PHA quantification, 20 mL of each *P. putida* strain grown in PHA production conditions (see 2.1 section) for 24 h were centrifuged for 30 min at 3,200 ×*g*. Cells were washed once with 0.85% NaCl and lyophilized for 24 h. Lyophilized pellets were weighed to obtain the cell dry weight (CDW) of total biomass. PHA monomer composition and PHA content were determined by Gas Chromatography-Mass Spectrometry (GC-MS) of the methanolysed polyester (Braunegg et al., 1978; Revelles et al., 2016; Manoli et al., 2022). In each condition, at least two independent biological replicates were performed. Where the statistical error was higher of 10%, four biological replicates were performed. During the methanolysis process, two technical replicates were included for each biological sample. 2–5 mg of lyophilized cells were resuspended in 2 mL of methanol acidified with 3% (v/v) H_2_SO_4_ for scl-PHA analysis, and resuspended in 2 mL of methanol containing 15% (v/v) H_2_SO_4_ for mcl-PHA analysis. 2 mL of chloroform containing 0.5 mg/mL 3-methylbenzoic acid was added to the samples as an internal standard. Samples were boiled in a screw-capped tube at 100°C for 4 or 5 h to assay scl-PHA or mcl-PHA, respectively. After cooling, the mixture was washed twice by adding 1 mL of distilled water, centrifuged for 10 min, and followed by the removal of the aqueous phase. The organic layer containing the resulting methyl ester of each monomer was analyzed by GC-MS using an Agilent 7890A GC equipped with a DB-5HT capillary column (30 m length, 0.25 mm internal diameter, 0.1 µm film thickness) and mass data were acquired and processed with an Agilent 5975C mass spectrometer. Samples (1 μL) of the organic phase were injected with helium as carrier gas at a ratio of 1:10 with 1 part sample to 10 parts helium, and the oven temperature was programmed to remain at 80°C for 2 min and then increased at a rate of 5°C/min up to 115°C for the efficient separation of peaks. The temperature of the injector was 250°C. Spectra were obtained as electron impacts with an ionizing energy for MS operation of 70 eV. Standard curves with known quantities of PHB (Sigma-Aldrich) or poly (3-hydroxyhexanoate-co-3-hydroxyoctanoate) (Bioplastech, Ltd.) dissolved in chloroform were used to calculate the monomer composition of the extracted polymers.

Monomer composition was also analysed by NMR. For these experiments, the used solvent was deuterated chloroform (chloroform-d 99.8%) that contains 0.03% (v/v) tetramethylsilane (TMS) (CDCl_3_) (ref. 225,789 from Sigma-Aldrich). Proton NMR spectra (^1^H-NMR) were recorded using a 90° pulse experiment under the following acquisition parameters: 128 scans with a fixed receiver gain value of 287, spectral width of 12.0164 ppm, 32,768 points in the time domain, and acquisition time of 2.27 s. COSY spectra were recorded using the standard Bruker sequence *cosygpqf*. Spectra were recorded under the following acquisition parameters: fixed receiver gain value of 1290, 128 scans and a spectral width of 13.0177 × 13.0177 ppm.

The 1D (1H), 2D Correlated Spectroscopy (COSY) NMR spectra of the extracted polymers were recorded on Bruker AV III 600 MHz spectrometer (Bruker, Rheinstetten, Germany) using a XI 600 MHz S3 5 mm probe with Z-gradient in CDCl_3_. The resulting NMR spectra were processed by Mestrelab MNova software (Version 14.2.3–29241). Phasing and baseline correction were manually completed.

According to ^1^H-NMR and COSY assays (Supplementary Figure S3), the signals assigned to the protons of the HB monomer are methyl at 1.27 ppm (4), methylene at 2.50 ppm (2) and methine at 5.25 ppm (3), and the protons assigned to the mcl-HA monomer are methyl at 0.90 ppm (9), methylene groups of the side chain at 1.28 and 1.59 ppm (8), methylene group at 2.50 ppm (6) and methine at 5.25 ppm (7).

### 2.7 Granule extraction and identification of key associated proteins

For granule extraction, cultures grown under PHA-producing conditions for 24 h as mentioned above were harvested by centrifugation for 20 min at 10,000 ×*g.* Cells were resuspended in 15 mM Tris-HCl pH 8.0 buffer and then disrupted twice using French Press (at ≈ 1 000 psi). PHB granules from pellets (resuspended in 15 mM Tris-HCl pH 8.0) were purified by two subsequent glycerol density gradient centrifugations at 18,000 ×*g* for 40 min. The first gradient consisted of 6 mL of sample layered over 3 mL of 85% glycerol and 6 mL of 50% glycerol. Granules were isolated after centrifugation with a Pasteur pipette at the glycerol 50%–85% interface. The second gradient consisted of 3 mL of granule fraction from the first gradient over 4 layers of 3 mL of glycerol at 85%, 80%, 60%, and 40% glycerol. Granules were isolated after centrifugation from the glycerol 40%–60% interface.

For granule-associated protein detection, independent granule isolations were normalized to the same PHB content by PHA quantification as explained above. Aliquots containing 22.5 μg of PHB from each extraction were run onto 12.5% SDS-PAGE gels and stained using BlueSafe (Nzytech).

For N-terminal sequencing of granule-associated proteins, these SDS-PAGE (12.5%) gels were transferred onto methanol activated-polyvinylidene fluoride (PVDF) membranes in a semidry transfer device (Biorad) soaked in transfer buffer (25 mM Tris, 192 mM glycine, 20% methanol, pH 8.3) for 1 h 15 min at 15 mV. The resulting transferred membranes were stained with Ponceau S stain (ThermoFisher), and the visible protein bands of selected proteins were subjected to N-terminal sequencing by Edman degradation in a protein sequencer (Applied Biosystems, Procise 494).

### 2.8 Microscopy assays

Cultures were routinely visualized with a 100× phase-contrast objective using an epifluorescence microscope Leica DM4B (Wetzlar, Germany) and images were taken with an attached camera (Leica DFC345 FX). Where needed a filter system L5 was used for GFP observation. In order to fix cells and achieve a correct superposition of images from the different channels, microscope slides were covered by a thin layer of 0.1% poly-L-lysine. Then, 5 µL of the cell suspension was deposited on the covered slide and immediately observed under microscopy.

For Transmission electron microscopy (TEM) experiments, *P. putida* cells previously grown during 24 h under M63 0.1 N minimal medium supplemented with 15 mM octanoate, were harvested and washed twice with 1 X PBS. Then, the cells were fixed for 1 h in 3% glutaraldehyde in PBS and washed twice with PBS. Samples were post-fixed in 1% osmium tetroxide and 0.8% potassium ferricyanide for 1 h at 4°C. Samples were washed with PBS prior to dehydration with an increasing gradient of ethanol (30%, 50%, 70%, 80%, 90% and 100%) of 10 min per step. Samples were embedded in LX112 resin and were polymerized for 48 h at 60°C. 60–80 nm sections were placed in copper grids of 75 mesh and stained with 5% uranyl acetate for 15 min and lead citrate for 3 min. Samples were viewed in a JEOL 1230 TEM and images were taken with a CMOS TVIPS 16 mp camera.

To determine the size of PHA granules from TEM micrographs, 50 cells of each engineered strain were selected, in which PHA granule diameters were measured using ImageJ software. In each case, 100 granules with sharp boundaries were selected and analyzed. Thus, to avoid measuring cells in different section plans, only granules from cells with a size of 0.9-1 x 2–2.2 µm (width x length) were considered (Galán et al., 2011).

## 3 Results

### 3.1 Generating a *Pseudomonas putida* chassis for customized constitutive scl-PHA production

The chassis applied in this work was the PHA-deficient strain (*P. putida* KT2440 Δ*ph*a, named as PP05_01). This lacks the native *pha* gene cluster, including *phaC1ZC2DFI* that encodes the two PHA synthases, the depolymerase, the transcriptional activator PhaD and the two phasins (including promoters and regulatory regions that drive the expression of *pha* genes) (Supplementary Figure S1 and Supplementary Table S3).

Phenotypic evaluation of the PP05_01 (Δ*pha*) strain confirmed that it does not produce PHA as determined microscopically by the complete lack of PHA granules when cultured in LB or 0.1 N M63 supplemented with 15 mM octanoate (Supplementary Figure S1) and by the lack of detectable PHA by GC-MS (Table 2). Growth of the PP05_01 strain was highly similar to wild type. Comparison of the two strains revealed lower relative CFUs in the wild type (Supplementary Figure S1). This was expected due to the allocation of metabolic resources towards PHA production in the wild type that were instead dedicated to biomass accumulation in the Δ*pha* strain (De Eugenio et al., 2010a; Manoli et al., 2022).

**TABLE 2:** PHA yield following 24 h of growth under the corresponding conditions using 0.1 N M63 minimal medium. The data correspond to the mean values and standard deviations of four biological replicates (with two technical replicates for the methanolysis analysis). Residual biomass indicates the biomass free of PHA. N.D.: not detected *data obtained from (Manoli et al., 2022).

Conditions	Strains	Total biomass (g/L)	PHB (%CDW)	PHA (g/L)	Residual biomass (g/L)
15 mM octanoate	PP05_01	0.56 ± 0.07*	N.D.*	N.D.*	0.60 ± 0.10*
	PP00_02	1.40 ± 0.09	69.39 ± 7.12	0.97 ± 0.09	0.48 ± 0.08
20 mM glucose	PP00_02	0.68 ± 0.05	48.96 ± 4.15	0.33 ± 0.04	0.35 ± 0.03
30 mM acetate	PP00_02	0.34	3.46 ± 0.46	0.01 ± 0.00	0.32 ± 0.00

Since PP05_01 strain will be the foundation for implanting an orthogonal, synthetic *phb* gene cluster designed for constitutive scl-PHA production, we firstly validated the scl-PHA production capacity when carrying an inducible monocopy system that allows the production of the PHA machinery in the presence of an inducer. Thus, we chromosomally inserted via mini Tn*5* transposon the P*trc*:*phaC* ^*Cn*^ *-phaA* ^*Cn*^ *-phaB* ^*Cn*^ cluster from *C*. *necator* driven by the P*trc* promoter inducible by IPTG. Two strains were obtained on wild type background and *pha* null, PP00_03 and PP00_02, respectively. By initial screening of PP00_03 using octanoate as carbon and energy source, we obtained 60% PHA/CDW, composed of 43% C4, 4% C6 and 53% C8 (data not shown). For the purpose of this study, the influence of different carbon sources under nitrogen limited conditions on PHA accumulation was evaluated in the resulting *pha* null background strain PP00_02 (Table 2). The best PHA accumulation was observed when a fatty acid precursor such as octanoic acid was used compared to glucose or acetate. In fact, PP00_02, lacking the *pha* cluster, reached high amounts of PHA accumulation with octanoate (e.g., nearly 70% PHA/CDW) and 50% of PHA/CDW under glucose conditions. Taking into account the toxicity effect of acetate and the low growth performance under these conditions (e.g., reaching up to 0.3 g/L total biomass), PP00_02 produced slight amounts of 3% PHA/CDW. Taken together, these experiments validated the PP05_01 chassis as modifiable to enable orthogonal constitutive PHA production.

### 3.2 Custom golden gate/MoClo assembly for constitutive *pha* expression constructs

For the installation of efficient constitutive PHA production machinery, we aimed to develop a modular, extensible system for the creation of PHA gene expression constructs that would be easy to deploy with the chassis strain. To this end, we adapted the Golden gate/MoClo assembly cloning technique to rapidly generate gene expression constructs organized in synthetic operons containing multiple transcription units with the minimum *phb* genes needed (Weber et al., 2011; Blázquez et al., 2023). The resulting synthetic *phb* clusters followed the modular structure shown in the synthetic *phb* orthogonalization pathway (Figure 2), which enabled interchangeability of genetic parts across modules as needed.

Since dosage of the different synthetic *phb* modules is crucial for proper functioning of the PHA machinery (Hiroe et al., 2012; Li et al., 2016; Li et al., 2017), we varied the strength of synthetic promoters driving the expression of these genes using low (14a) and medium (SynPro16 or SP16) strength constitutive promoters, previously validated in *P. putida* (Zobel et al., 2015; Tiso et al., 2016; Blázquez et al., 2023). During the strains’ construction, we considered the specificity of the *phb* module as a potential tool for diversification of the monomeric content and possibly different catalytic capacities of these enzymes in a heterologous chassis. For this, the wild type genes were obtained from bacteria able to produce different types of scl-PHA (i.e., *R. rubrum, P. pseudoalcaligenes* and *C. necator*). Numerous plasmid-based synthetic *phb* modules were generated and tested (Figure 2, Supplementary Table S1), each module contained the three minimal *phb* transcriptional units necessary for PHB production (i.e., PHA synthase, *phaC*; 3-ketoacyl-CoA thiolase, *phaA*; 3-ketoacyl-CoA reductase, *phaB*).

**FIGURE 3:**
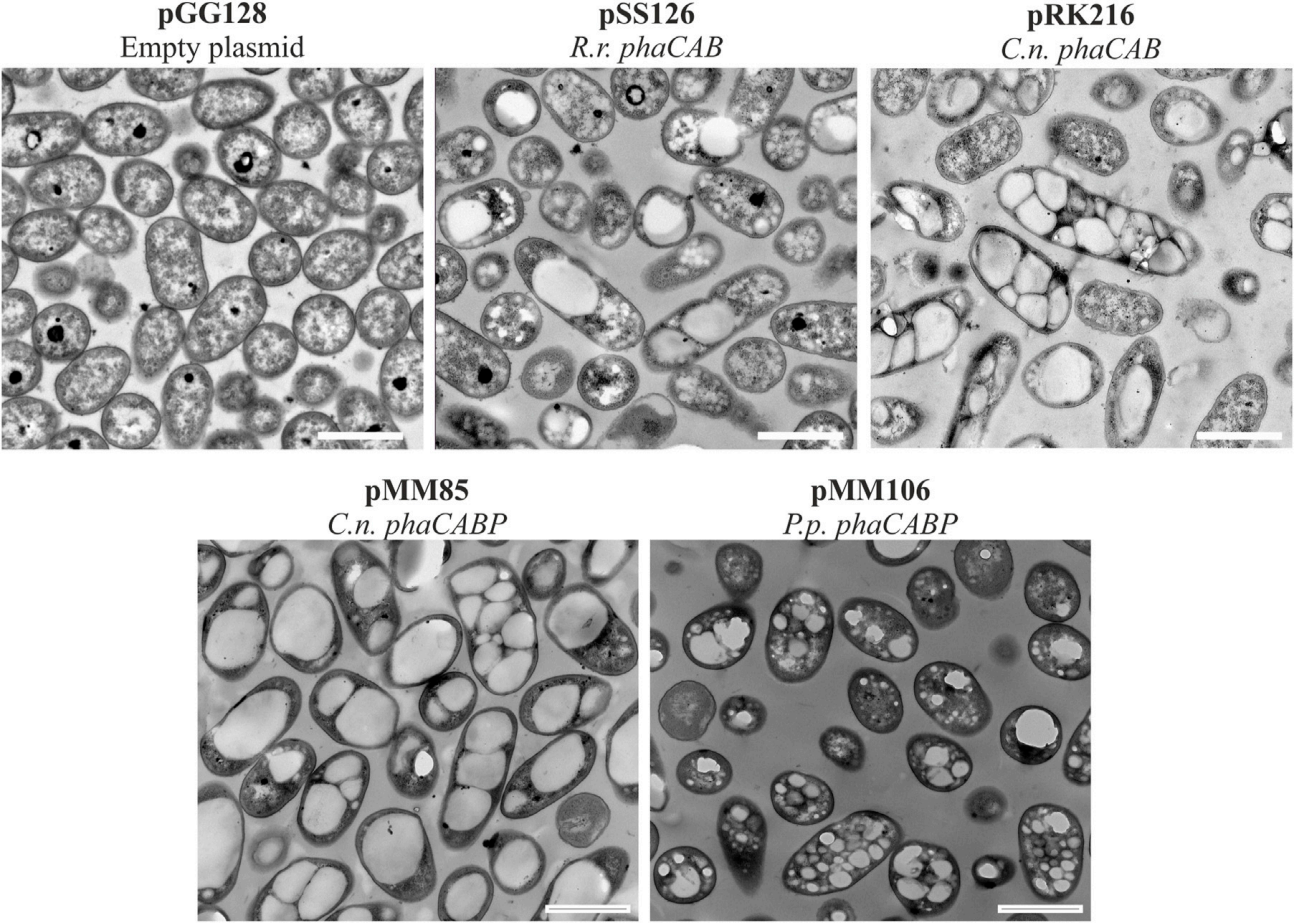
Transmission electron microscopy photos of heterologous PHA production. Pseudomonas putida PP05_01 strains expressing heterologous PHA machinery grown for 24 h in PHA production conditions with 15 mMoctanoate. Scale bars represent 1 μm. Abbreviations: R. r.: Rhodospirillum rubrum, C. n.: Cupriavidus necator, P. p.: Pseudomonas pseudoalcaligenes.

We assessed the production of scl-PHA (i.e., consisting of C4 or C5 monomers) and mcl-PHA (i.e., consisting of predominantly C6 and C8 monomers) following 24 h of growth in 0.1 N M63 minimal medium supplemented with 15 mM octanoate as the sole carbon source. The strain PP05_01 (pSS126), expressing the PHA machinery from *R. rubrum* under the low strength 14a constitutive promoter, yielded ∼25% CDW of PHB (Table 3). TEM images of PP05_01 (pSS126) strain revealed that most cells contained a single PHB granule occupying a large proportion of the cytoplasm (Figure 3). To improve the granule stability, in the same construct the three phasins from *R. rubrum* (i.e., A3283^*Rr*^, A2817^*Rr*^, A2111^*Rr*^) were additionally expressed under the SynPro16 promoter generating the PP05_01 (pRK182) strain. However, no major effect on the overall PHA production properties were observed by yielding 25% PHB/CDW (Table 3).

**TABLE 3:** Properties of heterologous PHA production in *Pseudomonas putida* chassis strain. GC-MS analysis of PHA content in 0.1 N M63 minimal medium supplemented with 15 mM octanoate for 24 h. The data correspond to the mean values and standard deviations of at least two independent biological replicates. TR.: Traces; N.D.: not detected.

Strain	Total CDW (g/L)	PHA (%CDW)	PHA (g/L)	Residual biomass (g/L)	%C4	%C6	%C8
KT2440 (pGG128; empty plasmid)	1.42 ± 0.01	61.73 ± 1.25	0.88 ± 0.01	0.54 ± 0.02	N.D.	7.11 ± 0.14	92.89 ± 0.14
PP05_01 (pGG128; empty plasmid)	0.43 ± 0.05	N.D.	N.D.	0.43 ± 0.05	N.D.	N.D.	N.D.
PP05_01 (pSS126; *R.r. phaCAB*)	0.67 ± 0.01	23.11 ± 1.04	0.15 ± 0.00	0.51 ± 0.02	99.57 ± 0.07	<0.5	<0.5
PP05_01 (pRK182; *R.r. phaCABP*)	0.60 ± 0.03	25.37 ± 1.59	0.15 ± 0.02	0.45 ± 0.01	100.00	TR.	TR.
PP05_01 (pRK216; *C.n. phaCAB*)	0.87 ± 0.04	47.13 ± 2.83	0.41 ± 0.05	0.46 ± 0.00	99.87 ± 0.02	<0.5	<0.5
PP05_01 (pMM85; *C.n. phaCABP*)	1.32 ± 0.10	84.31 ± 0.12	1.11 ± 0.08	0.21 ± 0.02	98.45 ± 0.54	0.98 ± 0.32	0.56 ± 0.21
PP05_01 (pMM106; *P.p. phaCABP*)	0.68 ± 0.02	44.90 ± 0.08	0.30 ± 0.01	0.37 ± 0.01	100.00	TR.	TR.

Similarly, the PHA machinery of *P. pseudoalcaligenes* (pMM106) and *C. necator* (pMM85) were expressed under the 14a constitutive promoter reaching 45%–84% PHA/CDW, respectively (Table 3; Figure 3). These were encouraging results, since we were able to obtain similar PHA content from *C. necator* genes expressed in a constitutive multicopy system compared to the inducible monocopy strain PP00_02 (Table 2). From TEM images, we could confirm that cells producing PHB generally contained multiple PHB granules that occupied the majority of the intracellular space (Figure 3). We also tested the influence of expressing the PHA machinery of *C. necator* under the moderate SynPro16 promoter (PP05_01 (pRK216)). However, assessing PP05_01 (pRK216) under the same growth scenario, there was no apparent improvement in the PHA production capabilities compared to the pMM85 plasmid (47% PHB/CDW, Table 3). In general, the PHA machinery constructs with SynPro16 promoter showed a more variable phenotype compared to 14a constitutive promoter. This can be also observed by the TEM images, where several cells harboring pRK216 did not produce PHA, leading to an overall decrease of the %PHA/CDW quantified by GC-MS analyses (Figure 3). Considering the unstableness issues raised by the SynPro16 promoter, in this study, we did not pursue the combination of the different genetic parts with this expression system.

As a product of a synthetic pathway involving four enzymes, PHA content relies heavily on the activity and relative ratios of these enzymes and the applied growth conditions. Thus, the best candidate strain harboring *C. necator* PHA machinery was tested for the feasibility to produce tailored PHA towards diverse personalized applications. For this purpose, several nutritional scenarios were planned for the production of copolymers (i.e., PHBV) in PP05_01 harboring pMM85 with the wild type *phb* ^*Cn*^ cassette. As listed in Table 4, a panel of PHBV copolymers was successfully obtained with varying C4:C5 compositions. In fact, co-feeding with 5 mM propionic acid yielded 35% of PHA/CDW with 3% C5 monomeric composition while 1 mM undecenoic acid co-feeding yielded 44% PHA/CDW with 19% of C5. Overall, we demonstrated the successful deployment of custom assembled plasmids to produce à la carte PHB/PHBV polymers in the engineered *P. putida* strains.

**TABLE 4:** PHA yield of PP05_01 harboring pMM85 (*C.n. phaCABP*) following 24 h growth with 0.1 N M63 supplemented with 20 mM glucose and co-fed with the indicated odd length fatty acids. The data correspond to the mean values and standard deviations of two independent biological replicates.

Conditions	Total biomass (g/L)	PHA (%CDW)	PHA (g/L)	Residual biomass (g/L)	%C4	%C5
not-cofed	0.97 ± 0.04	39.04 ± 3.09	0.38 ± 0.04	0.59 ± 0.03	100	N.D.
1 mM propionic	0.95 ± 0.03	33.00 ± 1.05	0.31 ± 0.00	0.64 ± 0.03	99.41 ± 0.01	0.59 ± 0.01
5 mM propionic	0.98 ± 0.08	34.61 ± 2.72	0.34 ± 0.05	0.64 ± 0.02	96.91 ± 0.61	3.09 ± 0.61
1 mM undecenoic	1.08 ± 0.12	44.26 ± 2.83	0.48 ± 0.08	0.60 ± 0.04	81.36 ± 4.52	18.64 ± 4.52

### 3.3 Phenotypic evaluation of chromosomally integrated PHB constructs

For scale up processing the ideal strain would not rely on antibiotic resistance for plasmid maintenance nor the use of an inducer for expression of the heterologous cassette. Additionally, the chromosomic PHA machinery integration would result to a more stable and homogeneous phenotype compared to plasmid. For this reason, a strain was generated to efficiently produce PHB in a monocopy constitutive expression system inserted in the *P. putida* chromosome. As a starting point, the *phb* cassette from *C. necator* was specifically inserted into the *att*Tn*7* loci of *P. putida* PP05_01, generating the PP05_16 and PP05_12 strains (Table 1). PP05_16 strain contained the four genes necessary to drive PHB synthesis under the low strength constitutive promoter 14a and PP05_12 did not contain the PhaP^Cn^ phasin (please refer to Table 1 and (Supplementary Table S1) for strains, plasmids and genomic information).

As expected, under the same growth conditions supplemented with 15 mM octanoate, the monocopy expression of the *phb* ^*Cn*^ cassette led to a decrease in percent PHA/CDW accumulation (Table 5) that was also reflected in a decrease in total biomass (12%–15% by PP05_12 and PP05_16 *versus* 84% with pMM85 plasmid). To improve on PHB productivity observed in PP05_12 and PP05_16, the PP01_02 strain was generated. PP01_02 harbored the same *phb* cassette as PP05_12 in the *att*Tn*7* chromosomic locus but with the *phaC* ^*Cn*^ synthase under control of a stronger 14f promoter (Table 5; Zobel et al., 2015). PP01_02 resulted in 69% PHA/CDW accumulation, which by NMR quantification we confirmed that 92% of the produced polymer was C4 and 8% C6 monomer (Supplementary Figure S3). Altogether, we successfully obtained a battery of chromosomic integrated PHB constructs that resulted in tuned PHA productivities (e.g., from 12%–69% PHA/CDW). These observations strongly suggested the impact of *pha* synthase dosage on PHA yield and monomeric composition.

**TABLE 5:** PHA yield in PP05_01 modified strain following 24 h growth with 0.1 N M63 supplemented with 15 mM octanoate as the sole carbon source. N.D.: not detected. Mean values and standard deviations of at least two independent biological replicates are shown. *Data derived from NMR quantification.

Plasmid	Total biomass (g/L)	PHA (%CDW)	PHA (g/L)	Residual biomass (g/L)	%C4	%C6	%C8
PP05_01 (pMM85)	1.32 ± 0.10	84.31 ± 0.12	1.11 ± 0.08	0.21 ± 0.02	98.45 ± 0.54	0.98 ± 0.32	0.56 ± 0.21
PP05_16	0.69 ± 0.02	14.56 ± 1.16	0.10 ± 0.01	0.59 ± 0.01	100.00	N.D.	N.D.
PP05_12	0.63 ± 0.02	11.78 ± 0.97	0.07 ± 0.01	0.56 ± 0.01	100.00	N.D.	N.D.
PP05_12 (pMM194)	0.62 ± 0.01	15.10 ± 1.81	0.09 ± 0.01	0.53 ± 0.01	100.00	N.D.	N.D.
PP01_02	1.02 ± 0.04	68.51 ± 5.78	0.70 ± 0.06	0.32 ± 0.06	92*	8*	N.D.
PP05_15	1.07 ± 0.05	63.47 ± 4.86	0.68 ± 0.04	0.39 ± 0.07	95.88 ± 1.25	2.80 ± 0.79	1.31 ± 0.49
PP05_15 (pMM194)	1.00 ± 0.05	65.29 ± 1.68	0.65 ± 0.05	0.35 ± 0.00	97.82 ± 0.06	1.51 ± 0.01	0.67 ± 0.07

### 3.4 Impact of PHA synthase dosage on number and size distribution of granules and the identification of a granule-associated heat shock protein

It is well known that PHA synthesis is tightly controlled by a number of regulatory networks that govern PHA content, granule size and distribution in cells (recently reviewed by (Mitra et al., 2022)). However, the heterologously produced phasins in engineered strains did not show an obvious influence over PHA production properties or granule size (Table 3; Figure 3).

**FIGURE 4:**
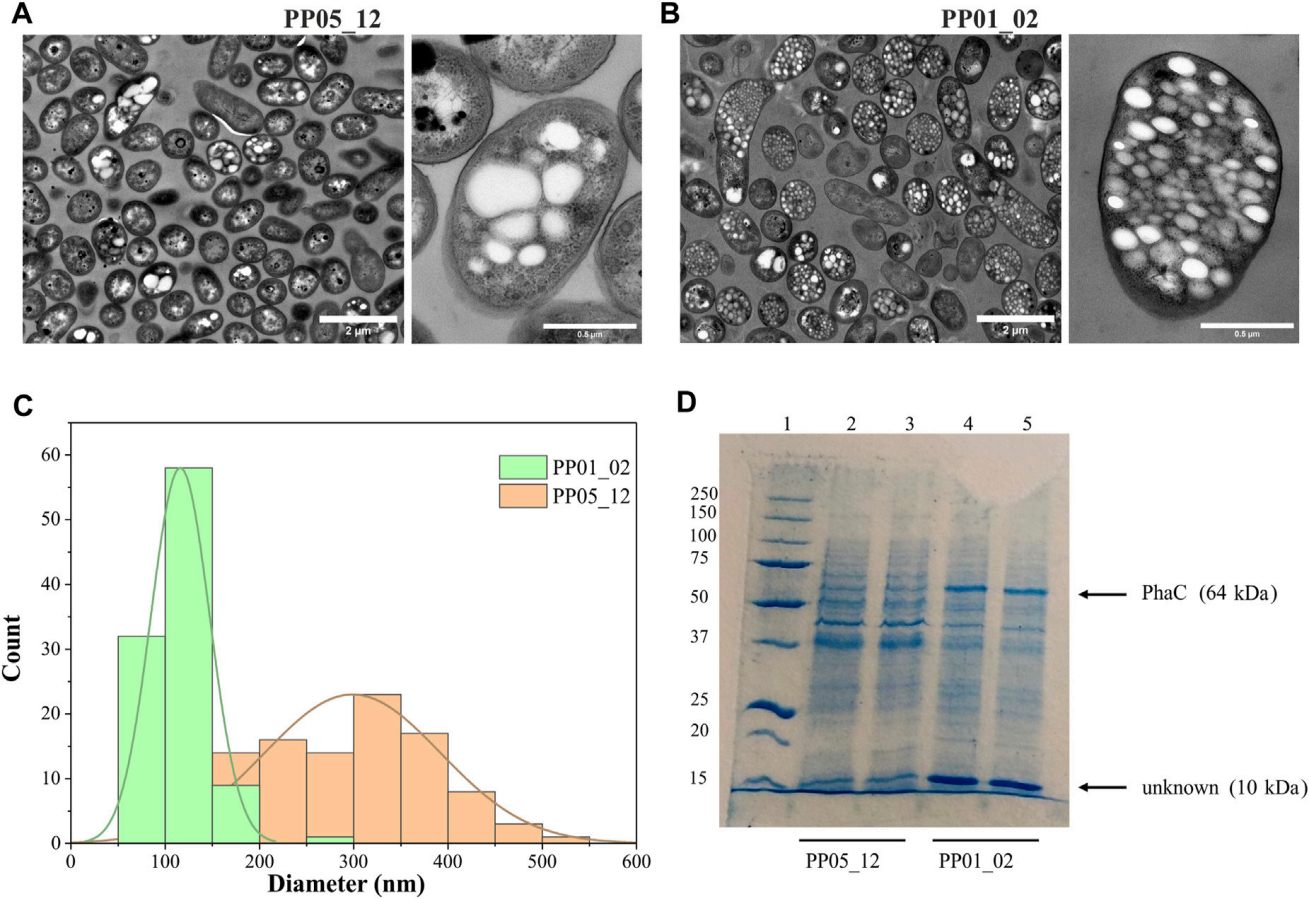
Impact of synthase dosage on granule number and size. **(A, B)**. TEM images of PP01_02 and PP05_12, respectively under standard PHA accumulation conditions supplemented with 15 mM octanoate. **(C)**. Granule size distribution obtained from 100 granules measured from TEM images using ImageJ software, PP01_02 (green) and PP05_12 (orange). **(D)**. SDS - PAGE gels of granule extractions. Lane 1 MW marker; lanes 2–3: PP05_12 independently extracted PHB granules; lanes 4–5: PP01_02 independently extracted PHB granules. MW of the corresponding GAPs are indicated. Precision Plus Protein Standards (Biorad) was used as a molecular weight marker using Tris-Glycine 4%–20% conditions.

To elucidate other factors that might impact granule number and size (i.e., synthase dosage), TEM microscopic photos were taken after 24 h of growth using 15 mM octanoate as the sole carbon and energy source. For this, PP05_12 and PP01_02 strains were used, expressing the *pha* synthase under the control of 14a (low strength) and 14f (high strength) promoters, respectively. Figure 4 shows that low synthase dosage in the PP05_12 strain, generated a heterogeneous cell population, with several cells without PHB inclusions and others with few, but large PHB granules. However, high synthase doses in the PP01_02 strain resulted in a more homogeneous population of cells that contained numerous smaller granules. Granule size analysis of the PP05_12 strain showed a broader size distribution with an average size of 300 nm, while PP01_02 strain had a narrower size distribution with a smaller average size of 180 nm (Figure 4C).

To further confirm the relative increase in PhaC synthase expression, granule preparations were extracted and run on SDS-PAGE to estimate GAPs present on the granule surface (Figure 4D). To ascertain that the same granule quantity was run on the gels, methanolysis of granule preparations were performed to quantify the actual amount of PHB in each preparation. The control experiments with the empty plasmid may be found in the Supplementary Figure S2. As expected, PP05_01 (pGG128) strain did not produce any detectable PHA and, thus, no granule formation was visible in the SDS-PAGE gels (Supplementary Figure S2). Comparing with the control band pattern, the presence of acetoacetyl-CoA-reductase PhaB (26 kDa) and acetyl-CoA-transferase PhaA (40 kDa) in both strains’ granule preparation resulted challenging. This could be explained to some extent by their low abundance in the granule surface since they were expressed under the low 14a promoter’s strength. However, as expected, the PP01_02 strain with a higher strength promoter for PhaC synthase (64 kDa) showed higher levels of PhaC protein than PP05_12 (Figure 4D). N-terminal sequencing of the 64 kDa band confirmed that this corresponded to PhaC.

In this granule preparations, we could observe a repeated pattern of a co-increased protein dosage of approximately 10 kDa protein together with the increase in PhaC^Cn^. Interestingly, this low molecular weight protein was also observed in the granules’ extraction harboring the PHA machinery from *R. rubrum* (Supplementary Figure S2). To elucidate the identity of this unknown protein, we performed N-terminal sequencing. A protein BLAST of “TTAFSLAPLF” against the *P. putida* KT2440 proteome revealed the presence of the small heat shock protein, lbpA (PP_1982).

### 3.5 Involvement of inclusion body protein IbpA in PHA production

Small heat-shock proteins (sHSP), are characterized by a molecular mass of 12–43 kDa, and function as ubiquitous and diverse molecular chaperones that prevent protein aggregation under heat shock conditions. Two sHSP from *E. coli*, IbpA/B have been previously reported to bind to the inclusion bodies of recombinant proteins (Han et al., 2006). IbpA is also considered a stress-related chaperone with an intrinsic holdase activity. This ATP-independent holding function allows them to bind to denatured and partly unfolded proteins under stress conditions. The proteins bound to sHSPs are maintained in a refolding-competent state and are thereby protected from irreversible aggregation (Han et al., 2006; Roy et al., 2014).

**FIGURE 5:**
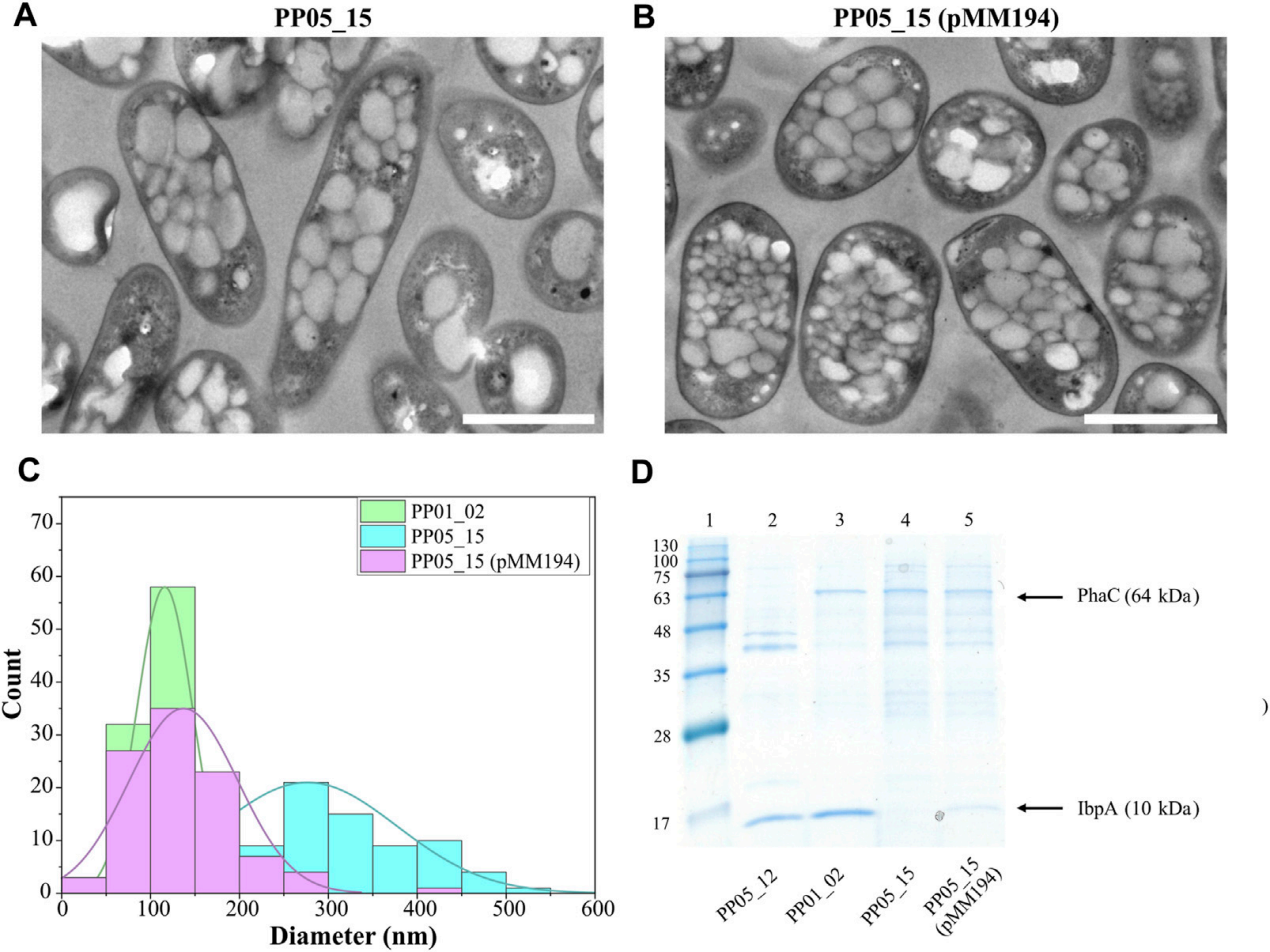
Impact of IbpA deletion on granule number and size. **(A, B)**. TEM images of PP05_15 and PP05_15 (pMM194), respectively under standard PHA accumulation conditions supplemented with 15 mM octanoate. **(C)**. Granule size distribution obtained from 100 granules measured from TEM images using ImageJ software, in green PP01_02, blue PP05_15 and purple PP05_15 (pMM194). Scale bars represent 1 µm. **(D)**. SDS - PAGE gels of granule extractions. Lane 1 MW marker; lanes 2: PP05_12; lane 3: PP01_02; lane 4: PP05_15; lane 5: PP05_15 (pMM194). The MW of the corresponding GAPs are indicated. BlueStar Prestained protein marker (Nippon Genetics) was used as a molecular weight marker using Tris-Glycine 4%–20% conditions.

To assess the involvement of IbpA on bacterial PHA machinery, an *ibpA* deletion mutant was constructed in the PP01_02 background, generating strain PP05_15. Phenotypic evaluation of PP05_15 showed no major impact on the growth capacity at 30°C compared to the parental PP01_02 strain (e.g., similar residual biomass, Table 5). These results are in agreement with previous observations to *ibpA* deletion mutant in KT2440, where the growth was only significantly affected at 40°C (Krajewski et al., 2013). Looking at the PHA profile properties, PP05_15 revealed no major differences concerning the PHA accumulation profile compared with the parental strain, PP01_02; 63.5% PHA/CDW *versus* 68.5% PHA/CDW, respectively (Table 5). These observations are in accordance with the recombinant *E. coli* IbpA/B null strain behavior, which also slightly affected PHA production, when carried the *C. necator* PHA machinery (Han et al., 2006). To look closer on the involvement of the IbpA deletion on granule size distribution, TEM analyses were performed (Figure 5A). In fact, PP05_15 strain showed a broader granule size distribution with an average size of about 300 nm compared with 180 nm of PP01_02 (Figure 5C). Finally, the influence of IbpA over other granule-associated proteins was analyzed by granule extraction assay (Figure 5D). As expected for granules extracted from the *ibpA* null (PP05_15) strain, the 10 kDa band corresponding to IbpA disappeared, while showing little change in the other bands corresponding to GAPs involved in PHA production (i.e., PhaC). It is worth mentioning that during the extraction procedure granules from the PP05_15 strain appeared to be more aggregated and adherent compared to granules from PP01_02. This aspect could be explained, at least to some extent, from the deletion of IbpA, which could lead to higher protein aggregation.

Complementation assays with pMM194 plasmid were performed by introducing the *ibpA* gene expressed by the constitutive 14a promoter:BCD2 element. Similar PHA production properties were obtained by the complemented strains PP05_12 (pMM194) and PP05_15 (pMM194), reaching 15% and 65% PHA/CDW, similar to the parental strains (Table 5). This could be explained to some extent by the low dosage of complemented IbpA. We cannot discard that higher IbpA dosage could lead to higher PHA content to the engineered strains. Even though no major effect was observed by PHA production, TEM images revealed that PP05_15 (pMM194) successfully reverted the granule formation pattern, with a clear tendency towards smaller granule size compared to PP01_02 strain (Figures 5B, C). Additionally, granule extraction assays confirmed that the presence of IbpA by pMM194 resulted in less granule aggregation (data not shown) and a re-appearance of the IbpA band to the SDS-PAGE (Figure 5D).

**FIGURE 6:**
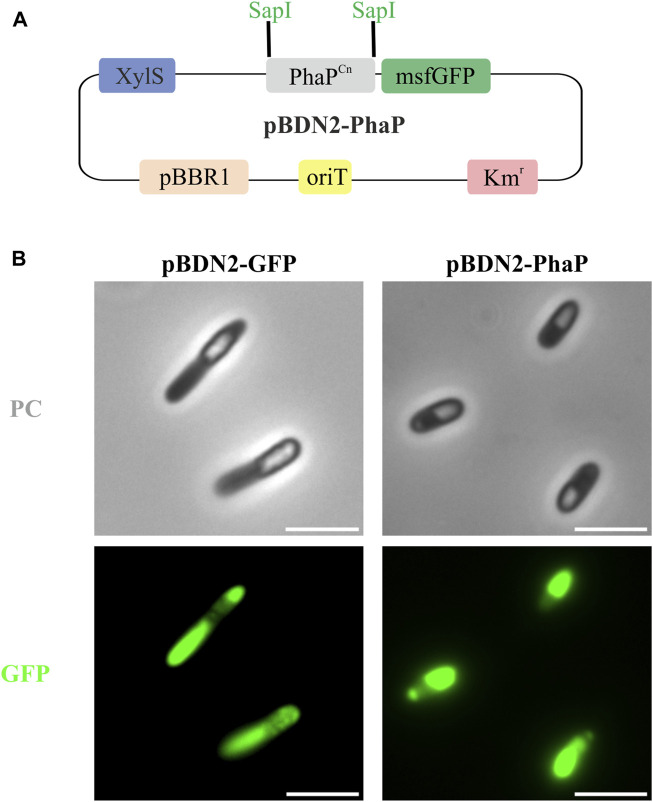
Co-localization experiments in the scl-PHA chassis (PP01_02). **(A)**. Schematic representation of pBDN2-PhaP vector. **(B)**. Fluorescence images with phase contrast (PC) amd GFP, left panel PP01_02 (pBDN2-GFP) negative control of binding, the strain that expresses the msf-GFP gene and right panel PP01_02 (pBDN2-PhaP) that expresses the PhaP1 from *Cupriavidus necator*. The scale bar corresponds to 3 µm.

IbpA/B seem to exhibit several different functions depending on physiological conditions, among them it has been ascribed that these phasin-like proteins might function as a phase stabilizer at the interface of hydrophilic cytoplasm and hydrophobic PHB granules when this polymer is heterologously produced in *E. coli* (Han et al., 2006). To elucidate if the presence of IbpA in the granule could inhibit the binding of other GAPs, we tested the binding of the PhaP phasin from *C. necator* to scl-PHA granules*.* For this, PhaP1^*Cn*^ was fused to the green fluorescent protein (msf-GFP) and heterologously expressed in the PP01_02 strain (Figure 6A). For the co-localization experiments, as expected, the control PP01_02 strain harboring (pBDN2-GFP), empty plasmid including only the msf-GFP, showed a diffuse cytoplasmic fluorescence surrounding the scl-PHA granules (Figure 6B). This observation confirmed the non-binding affinity of msf-GFP towards scl-PHA. As anticipated, the scl-family phasin (e.g., PhaP1^*Cn*^ in pBDN2-PhaP plasmid) showed a co-localized fluorescence to the polymer, thus, maintaining its ability to bind to scl-PHA even in the presence of IbpA (Figure 6B).

## 4 Discussion

### 4.1 *P. putida* as a PHA production chassis

*Pseudomonas putida* is a model bacterium for mcl-PHA production with a complex regulatory system driving the expression of genes encoding the PHA machinery. When grown on fatty acids, transcription of *pha* locus genes is augmented when compared to growth on simple carbon sources (De Eugenio et al., 2010b; Wang and Nomura, 2010; Manoli et al., 2020). Additionally, the Crc catabolite repression regulator influences transcription of *phaC1* such that its transcription is inhibited in balanced carbon/nitrogen conditions (La Rosa et al., 2014). While other global transcriptional factors, including RpoS, PsrA and GacS/GacA also influence transcription of genes in the *pha* locus, though their precise roles remain to be fully elucidated (De Eugenio et al., 2010b; Mezzina et al., 2021). In fact, from previous works from our group, we showed a complex interactome among key components of PHA production (i.e., PhaF-PhaD) (Tarazona et al., 2020). However, a deletion of the entire *pha* locus disengages these regulatory functions from the native production of mcl-PHA, making orthogonal PHA production independent of cellular regulators of gene expression. Nevertheless, we cannot discard the involvement of other cellular components during the heterologously expressed scl-PHA machinery. A full *pha* locus deletion chassis strain can then serve to host heterologous *pha* expression constructs. This allows for *à la carte* production of bespoke PHAs in *P. putida* using custom expression constructs coupled with growth of the resulting strain on any number of carbon sources.

There have been numerous examples of the production of orthogonal PHA in *pha* null *Pseudomonas* through the heterologous expression of *pha* genes. These include the expression of *phaC* synthase genes from scl-/mcl-PHA producing *C. necator*, *Rhodobacter sphaeroides*, *Nocardia corrallina*, *Thiocystis violacea*, or various pseudomonads in Δ*pha* mutants of *P. putida* to generate scl- and mcl-PHAs with unique compositions (Huisman et al., 1991; Preusting et al., 1993; Timm et al., 1994; Lee et al., 1995; Dennis et al., 1998; Matsusaki et al., 1998; Clemente et al., 2000; Ouyang et al., 2007). Another study created a *P. putida* KT2440 *phaC1* ^-^, Δ*phaZ*, *phaC2* ^-^ *Ω* interposon mutant and used this Δ*pha* strain as a host for the functional screening of soil metagenomic cosmid clones to identify novel PHA synthases (Cheng and Charles, 2016). Our work expands upon these previous investigations by generating a sequence-confirmed *P. putida* KT2440 Δ*pha* locus chassis, PP05_01 strain coupled with the advantage of using a highly flexible assembly cloning system for the generation of custom, stable and constitutively produced scl- and mcl-PHAs.

Pseudomonads can utilize direct PHA precursor pathways to convert fatty acids and PHA unrelated carbon sources (i.e., acetate, ethanol, glycerol, sugars, etc.) via *ß*-oxidation and *de novo* synthesis of fatty acids, respectively, into various (*R*)-3-hydroxyacyl-CoAs (Figure 1). In contrast, *R. rubrum, P. pseudoalcaligenes,* and *C. necator* are limited in their ability to channel fatty acid metabolites into PHA and instead PHA production relies upon the availability of acetyl-CoA derived from the catabolism of carbon sources (Senior and Dawes, 1971; Budde et al., 2010). The creation of the PP05_01 chassis strain lacking the entire *pha* locus allows for the decoupling of the natural cycle of mcl-PHA production and consumption that is an integral part of energy apportionment and central carbon flux in *P. putida* (Escapa et al., 2012). As mentioned above, mcl-PHA production in *P. putida* is connected to central and peripheral metabolic pathways. The PP05_01 chassis is not subject to catabolite repression of PHA machinery expression and thus, avoids the dependence on certain carbon sources for PHA production. Generally, a nutritional imbalance (i.e., excess carbon and/or the limitation of nutrients such as nitrogen) favors PHA production in *P. putida* (Madison and Huisman, 1999). This imbalance is most significant for PHA production when substrates other than fatty acids are used as carbon sources (Sun et al., 2007; Wang and Nomura, 2010; Follonier et al., 2011). In the presence of fatty acids, nitrogen limitation is not necessary for PHA production, yet greatly improves PHA yields (Poblete-Castro et al., 2012). Notwithstanding, for our studies in the majority of cases, PHA production was carried out in nitrogen limited conditions with octanoate as the sole carbon source.

### 4.2 Expanding the range of PHA production in *P. putida*

The modular and hierarchical nature of biological designs reveals new possibilities for the development of rational and standardized mechanisms in order to improve the engineering process of specific biological solutions. DNA assembly is a widely used method to build synthetic genetic circuits. Traditionally, these cloning strategies were performed by digestion and ligation of DNA fragments using BioBricks or Gibson assembly. However, these approaches require specific designs for each step that can hamper complete standardization and parts reuse. Modular Cloning (MoClo) methodology has emerged as a powerful tool for standardizing the assembly of genetic parts. MoClo is based on Golden gate cloning, which allows simultaneous and directional assembly of multiple DNA parts.

PHA polyesters can be derived from over 150 different (*R*)-3-hydroxyalkanoic acid monomers, giving rise to a huge variety of physical and mechanical properties in the resulting polymers (Steinbüchel and Valentin, 1995; Hazer and Steinbüchel, 2007). Production of orthogonal PHAs in *P. putida* greatly expands the envelope of polymer diversity. Due to its metabolic flexibility and ability to use a variety of substrates, such as fatty acids or aromatic compounds, it is expected that our *P. putida* toolkit can be exploited to generate new and useful PHAs (Linger et al., 2014; Blázquez et al., 2023).

By using Golden gate/MoClo technology, we demonstrated the successful production of PHB and PHBV copolymers in *P. putida* PP05_01 through the expression of heterologous PHB machinery from *R. rubrum, P. pseudoalcaligenes* or *C. necator*. Indeed, since the synthetic *phb* clusters used in this study followed the same brick structure, this could enable the interchangeability of the genetic parts across the modules, if needed. Thus, we could discover the best synthetic parts’ (i.e., promoter, RBS, CDS) combination for most optimal PHA production. Additionally, the involvement of phasins in PHA accumulation and granule stability was studied, suggesting that the presence of these proteins in a heterologous host is not crucial. The absence or presence of phasins did not demonstrate significant differences in PHA accumulation and granule formation. However, we observed that the dosage of PhaC in the chromosome integrated constructs significantly impacted PHA production in several ways. In this sense, we identified interesting patterns: i) higher scl-PHA production, ii) production of a panel of PHAs with increased mcl-PHA (predominantly C6) composition (i.e. 90% C4 and approximately 10% mcl-PHA, iii) more numerous and smaller size granules, and iv) that the presence of the small heat shock protein IbpA augments heterologous PHA production.

### 4.3 IbpA is an additional player in PHB granule stability

IbpA/B belong to the alpha-crystalline type small heat-shock proteins (sHSP) with a molecular mass of 12–43 kDa, and are known to act as holding chaperones (Krajewski et al., 2013; Roy et al., 2014). Most chaperones possess intrinsic holdase activity, where the ATP-independent holding function is used to bind unstable proteins and prevent the formation of dysfunctional aggregates. Then, misfolded proteins can be transferred from holdase chaperones to downstream ATP-dependent chaperones. These use energy from ATP hydrolysis to power conformational changes in the chaperone, which promotes unfolding, refolding, or translocation of bound substrate proteins as part of their processing. Therefore, the combined action of molecular chaperones may increase the cellular pool of native proteins while minimizing inactive proteins and potentially harmful protein aggregates (Jewett and Shea, 2006).

Indeed, two sHSP from *E. coli*, IbpA/B have been previously reported to bind to the inclusion bodies of recombinant proteins. Han and collaborators demonstrated that a recombinant *E. coli* IbpA/B deletion mutant led to significant changes in PHB granule morphology, whereby they were shown to become distorted and wrinkled (Han et al., 2006). Therefore, it was suggested that in the absence of IbpA/B, PHB granules were expected to bind more cytosolic proteins in a non-specific manner compared to the parental strain. For this, IbpA/B in *E. coli* were proposed to act as phasin-like proteins that function as a phase stabilizer at the interface of the hydrophobic PHB granules and the hydrophilic cytoplasm (Han et al., 2006).

In this work, we constructed a viable IbpA null, PP05_15 strain. Even though the phenotypic evaluation revealed no major changes in PHA production, differences were observed in granule size distribution. Indeed, in the absence of IbpA, the tendency was towards overall larger granules compared to parental PP01_02 strain. Interestingly, this granule size distribution of PP05_15 is quite similar to PP05_12 (containing low PhaC synthase expression), suggesting that the deletion of IbpA may decrease the effective level or function of PhaC. We did not observe an obvious lower PhaC synthase level in the granule extracts of PP05_15 compared to PP01_02, indicating that the IbpA either modulates the function of PhaC by allowing its proper folding or operates on granule size in a PhaC-independent manner. Whether or not the PhaC/other granule-associated proteins could be partially aggregated in the PP05_15 strain was not determined, yet our findings confirmed the importance of IbpA protein in granule size determination and GAPs localization.

Altogether, in this study we demonstrated that *P. putida* optimized cell factories can be used for the production of tailored scl-PHA. Our results also suggest that native mcl-PHA regulatory network might be different to that of orthogonal scl-PHA system and we cannot discard the involvement of non-envisaged players such as IbpA.

## Data Availability

The raw data supporting the conclusion of this article will be made available by the authors, without undue reservation.
