# Supplementary material for: Heterologous constitutive production of short-chain-length polyhydroxyalkanoates in Pseudomonas putida KT2440: the involvement of IbpA inclusion body protein
Source: Front Bioeng Biotechnol. 2023 Nov 1;11:1275036. doi: 10.3389/fbioe.2023.1275036 (PMC10646324; doi:10.3389/fbioe.2023.1275036)
Supplement: Supplementary file 1 [file DataSheet1.docx]

Supplementary Material

**TABLE S1.** Plasmids used in this study

| Plasmids | Relevant characteristics | Reference/Genbank or Addgene accession number |
| --- | --- | --- |
| **Vectors not related to Golden gate cloning** | | |
| pEMG | Km^r^, *ori* R6K, *lacZα* with two flanking I‐*Sce*I sites | (Martínez-García and de Lorenzo, 2011a); JF965437 |
| pSW-I | Amp^r^, *ori* RK2, *xylS*, P*m*→*I-Sce*I (transcriptional fusion of I‐*Sce*I to P*m*) | (Martínez-García and de Lorenzo, 2011a) |
| pMM192 | Km^r^, *ori* R6K, pEMG derivative vector used for PP_1982 (*ibpA* gene) deletion | This work |
| pTnS-1 | Amp^r^, *ori* R6K, TnSABC+D operon | (Choi et al., 2005) |
| pTn*7*-M | Km^r^ Gm^r^, *ori* R6K, Tn*7*L and Tn*7*R extremes, standard multiple cloning site | (Zobel et al., 2015); KR920750 |
| pSEVA182 | Amp^r^, *ori* pUC, was used as receptor for the Golden standard Level 0 | SEVA team |
| pMAB26 | Km^r^, Ptrc promoter inducible with 1 mM IPTG, pCNB5 derivative plasmid containing *phaC^Cn^*, *phaA^Cn^* and *phaB*^Cn^ genes inserted in the mini-Tn*5* element | (Pais et al., 2014) |
| pBDN2 (pFB34) | Km^r^, *ori* pBBR1, GS cloning vector level 2. Contains the canthaxanthin marker flanked by SapI restriction sites, enables translational fusions with the msfGFP. Expression is under the control of the XylS/Pm system. | (Blanco et al., 2023) |
| pBDN2-GFP (pFB35) | Km^r^, *ori* pBBR1, pBDN2 derivative vector with a dummy sequence codifying for the amino acids MSGAG cloned at the SapI sites. Codifies for msfGFP with these 5 amino acids at the N-terminal sequence. | (Blanco et al., 2023) |
| pBDN2-PhaP (pFB36) | Km^r^, *ori* pBBR1, pBDN2 derivative vector with the *phaP* sequence cloned at the SapI sites. Codifies for PhaP-GFP. | (Blanco et al., 2023) |
|  |  |  |
| **Golden gate cloning vectors level 0** | | |
| pICH41295 | Sp/Sm^r^, *ori* pMB1/ColE1^‡^, Golden gate cloning vector level 0_ promoter region, position AD with l*acZa*, flanked by BpiI restriction sites | (Weber et al., 2011), 47997 |
| pICH41308 | Sp/Sm^r^, *ori* pMB1/ColE1^‡^, Golden gate cloning vector level 0_ coding sequence region position DG with l*acZa*, flanked by BpiI restriction sites | (Weber et al., 2011), 47998 |
| pICH41276 | Sp/Sm^r^, *ori* pMB1/ColE1^‡^, Golden gate cloning vector level 0_ terminator region position GI with l*acZa*, flanked by BpiI restriction sites | (Weber et al., 2011), 47994 |
| pSEVA182-BG25_AB | Amp^r^, *ori* pUC, Golden standard vector L0 pSEVA182 derivative, containing the 14f promoter, AB fusion sites flanked by BsaI restriction sites, | (Blázquez et al., 2023) |
| pSS15-BG28_AD | Sm^r^, *ori* pMB1/ColE1^‡^, pICH41295 derivative vector, containing 14a promoter-BCD2, AD fusion sites flanked by BsaI restriction sites | This work |
| pRK154_SynPro_AD | Sm^r^, *ori* pMB1/ColE1^‡^, pICH41295 derivative vector, λT0-SynPro16 promoter-RBS, AD fusion sites flanked by BsaI restriction sites | (Blázquez et al., 2023) |
| pSEVA182-BCD2_BD | Amp^r^, *ori* pUC, Golden standard vector L0 pSEVA182 derivative, containing the bicistronic BCD2 RBS sequence, flanked by BsaI restriction sites | (Blázquez et al., 2023) |
| pRK106-rnpBT1_GI | Sm^r^, *ori* pMB1/ColE1^‡^, pICH41276 derivative vector, rnpB-T1 terminator, GI fusion sites flanked by BsaI restriction sites | (Blázquez et al., 2023) |
| pRK107_rpoC_GI | Sm^r^, *ori* pMB1/ColE1^‡^, pICH41276 derivative vector, rpoC terminator, GI fusion sites flanked flanked by BsaI restriction sites | (Blázquez et al., 2023) |
| pRK108_T500_GI | Sm^r^, *ori* pMB1/ColE1^‡^, pICH41276 derivative vector, T500 terminator, GI fusion sites flanked flanked by BsaI restriction sites | (Blázquez et al., 2023) |
| pSS14_T1_GI | Sm^r^, *ori* pMB1/ColE1^‡^, pICH41276 derivative vector, λT1 terminator, GI fusion sites flanked by BsaI restriction sites | This work |
| pRK208-*C.n. phaA*_DG | Sp/Sm^r^, *ori* pMB1/ColE1^‡^, pICH41308 derivative vector containing the *phaA^Cn^* CDS, DG fusion sites flanked by BsaI restriction sites | This work |
| pRK209-*C.n. phaB1*_DG | Sp/Sm^r^, *ori* pMB1/ColE1^‡^, pICH41308 derivative vector containing the *phaB1^Cn^* CDS, DG fusion sites flanked by BsaI restriction sites | This work |
| pRK210-*C.n .phaC*_DG | Sp/Sm^r^, *ori* pMB1/ColE1^‡^, pICH41308 derivative vector containing the *phaC^Cn^* CDS, DG fusion sites flanked by BsaI restriction sites | This work |
| pMM70-*C.n. phaP1*_DG | Sp/Sm^r^, *ori* pMB1/ColE1^‡^, pICH41308 derivative vector containing the *phaP1^Cn^* CDS, DG fusion sites flanked by BsaI restriction sites | This work |
| pSS10-*R.r. phaC2*_DG | Sp/Sm^r^, *ori* pMB1/ColE1^‡^, pICH41308 derivative vector containing the A2413-*phaC2^Rr^* CDS, DG fusion sites flanked by BsaI restriction sites | This work |
| pSS13-*R.r. phaA*_DG | Sp/Sm^r^, *ori* pMB1/ColE1^‡^, pICH41308 derivative vector containing the A0274-*phaA^Rr^* CDS, DG fusion sites flanked by BsaI restriction sites | This work |
| pSS11-*R.r. phaB*_DG | Sp/Sm^r^, *ori* pMB1/ColE1^‡^, pICH41308 derivative vector containing the A0273-*phaB^Rr^* CDS, DG fusion sites flanked by BsaI restriction sites | This work |
| pRK151*-R.r. A2817*_DG | Sp/Sm^r^, *ori* pMB1/ColE1^‡^, pICH41308 derivative vector containing the A2817-phasin*^Rr^* CDS, DG fusion sites flanked by BsaI restriction sites | This work |
| pRK152-*R.r. A3282*_DG | Sp/Sm^r^, *ori* pMB1/ColE1^‡^, pICH41308 derivative vector containing the A3282-phasin*^Rr^* CDS, DG fusion sites flanked by BsaI restriction sites | This work |
| pRK153-*R.r. A2111* _DG | Sp/Sm^r^, *ori* pMB1/ColE1^‡^, pICH41308 derivative vector containing the A2111-phasin*^Rr^* CDS, DG fusion sites flanked by BsaI restriction sites | This work |
| pMM72-*P.p. phaC5*_DG | Sp/Sm^r^, *ori* pMB1/ColE1^‡^, pICH41308 derivative vector containing the *phaC5^Pp^* CDS, DG fusion sites flanked by BsaI restriction sites | This work |
| pMM71-*P.p. phaA3*_DG | Sp/Sm^r^, *ori* pMB1/ColE1^‡^, pICH41308 derivative vector containing the *phaA3^Pp^* CDS, DG fusion sites flanked by BsaI restriction sites | This work |
| pMM68-*P.p. phaB*_DG | Sp/Sm^r^, *ori* pMB1/ColE1^‡^, pICH41308 derivative vector containing the *phaB^Pp^* CDS, DG fusion sites flanked by BsaI restriction sites | This work |
| pMM69-*P.p. phaP*_DG | Sp/Sm^r^, *ori* pMB1/ColE1^‡^, pICH41308 derivative vector containing the *phaP^Pp^* CDS, DG fusion sites flanked by BsaI restriction sites | This work |
| pMM193-*P. p. ibpA*_DG | Sp/Sm^r^, *ori* pMB1/ColE1^‡^, pICH41308 derivative vector containing the *ibpA^Pp^* CDS, DG fusion sites flanked by BsaI restriction sites | This work |
| **Golden gate cloning vectors level 1** | | |
| pICH47732 | Amp^r^, *ori* ColE1^‡^/RK2, Golden gate cloning vector level 1 position 1 with *lacZa,* flanked by BsaI restriction sites | (Weber et al., 2011), 48000 |
| pICH47742 | Amp^r^, *ori* ColE1^‡^/RK2, Golden gate cloning vector level 1 position 2 with *lacZa,* flanked by BsaI restriction sites | (Weber et al., 2011), 48001 |
| pICH47751 | Amp^r^, *ori* ColE1^‡^/RK2, Golden gate cloning vector level 1 position 3 with *lacZa,* flanked by BsaI restriction sites | (Weber et al., 2011), 48002 |
| pICH47761 | Amp^r^, *ori* ColE1^‡^/RK2, Golden gate cloning vector level 1 position 4 with *lacZa,* flanked by BsaI restriction sites | (Weber et al., 2011), 48003 |
| pICH47772 | Amp^r^, *ori* ColE1^‡^/RK2, Golden gate cloning vector level 1 position 5 with *lacZa,* flanked by BsaI restriction sites | (Weber et al., 2011), 48004 |
| pICH41744 | Sp/Sm^r^, *ori* pMB1/ColE1^‡^, MoClo end-linker position 2*,* flanked by BsaI restriction sites | (Weber et al., 2011), 48017 |
| pICH41766 | Sp/Sm^r^, *ori* pMB1/ColE1^‡^, MoClo end-linker position 3*,* flanked by BsaI restriction sites | (Weber et al., 2011), 48018 |
| pSEVA23g19g1 | Km^r^, *ori* pBBR1, Golden standard vector level 1 position 1 with lacZa*,* flanked by BsaI restriction sites | (Blázquez et al., 2023) |
| pSEVA23g19g5 | Kmr, *ori* pBBR1, Golden standard vector level 1 position 5 with lacZa, flanked by BsaI restriction sites | (Blázquez et al., 2023) |
| pFB46 | Km^r^, *ori* pBBR1, pSEVA23g19g1 derivative vector. 14f:BCD2- *phaC1^Cn^*-*rnpB*T1; position 1, flanked by BpiI restriction sites | This work |
| pMM73 | Amp^r^, *ori* ColE1^‡^/RK2, pICH47732 derivative vector. 14a:BCD2-*phaC1^Cn^*-*rnpB*T1; position 1, flanked by BpiI restriction sites | This work |
| pMM74 | Amp^r^, *ori* ColE1^‡^/RK2, pICH47742 derivative vector. 14a:BCD2-*phaA^Cn^*-*rpoC* term; position 2, flanked by BpiI restriction sites | This work |
| pMM75 | Amp^r^, *ori* ColE1^‡^/RK2, pICH47751 derivative vector. 14a:BCD2-*phaB1^Cn^*-λT1; position 3, flanked by BpiI restriction sites | This work |
| pMM76 | Amp^r^, *ori* ColE1^‡^/RK2, pICH47761 derivative vector. 14a:BCD2-*phaP1^Cn^*-*rnpB*T1; position 4, flanked by BpiI restriction sites | This work |
| pL1F-1 *phaC1* | Amp^r^, *ori* ColE1^‡^/RK2, pICH47732 derivative vector. λT0-SynPro16-*phaC1^Cn^*-*rnpB*T1; position 1, flanked by BpiI restriction sites | (Blázquez et al., 2023) |
| pL1F-2 *phaA* | Amp^r^, *ori* ColE1^‡^/RK2, pICH47742 derivative vector. λT0-SynPro16-*phaA^Cn^*-*rpoC* term; position 2, flanked by BpiI restriction sites | (Blázquez et al., 2023) |
| pL1F-3 *phaB1* | Amp^r^, *ori* ColE1^‡^/RK2, pICH47751 derivative vector. λT0-SynPro16-*phaB1^Cn^*-T500; position 3, flanked by BpiI restriction sites | (Blázquez et al., 2023) |
| pMM78 | Amp^r^, *ori* ColE1^‡^/RK2, pICH47732 derivative vector. 14a:BCD2-*phaC5^Pp^*-*rnpB*T1; position 1, flanked by BpiI restriction sites | This work |
| pMM79 | Amp^r^, *ori* ColE1^‡^/RK2, pICH47742 derivative vector. 14a:BCD2-*phaA3^Pp^*-*rpoC* term; position 2, flanked by BpiI restriction sites | This work |
| pMM80 | Amp^r^, *ori* ColE1^‡^/RK2, pICH47751 derivative vector. 14a:BCD2-*phaB^Pp^*-λT1; position 3, flanked by BpiI restriction sites | This work |
| pMM81 | Amp^r^, *ori* ColE1^‡^/RK2, pICH47761 derivative vector. 14a:BCD2-*phaP1^Pp^*-*rnpB*T1; position 4, flanked by BpiI restriction sites | This work |
| pSS103 | Amp^r^, *ori* ColE1^‡^/RK2, pICH47732 derivative vector. 14a:BCD2-*phaC2^Pp^*-λT0; position 1, flanked by BpiI restriction sites | This work |
| pSS123 | Amp^r^, *ori* ColE1^‡^/RK2, pICH47742 derivative vector. 14a:BCD2-*phaA^Rr^*-λT0; position 2, flanked by BpiI restriction sites | This work |
| pSS124 | Amp^r^, *ori* ColE1^‡^/RK2, pICH47751 derivative vector. 14a:BCD2-*phaB^Rr^*-λT0; position 3, flanked by BpiI restriction sites | This work |
| pRK160 | Amp^r^, *ori* ColE1^‡^/RK2, pICH47751 derivative vector. λT0-SynPro16-A3282*^Rr^*-λT1; position 4, flanked by BpiI restriction sites | This work |
| pRK162 | Amp^r^, *ori* ColE1^‡^/RK2, pICH47751 derivative vector. λT0-SynPro16-A2817*^Rr^*-λT1; position 5, flanked by BpiI restriction sites | This work |
| pRK164 | Amp^r^, *ori* ColE1^‡^/RK2, pICH47751 derivative vector. λT0-SynPro16-A2111*^Rr^*-λT1; position 6, flanked by BpiI restriction sites | This work |
| pMM194 | Km^r^, *ori* pBBR1, pSEVA23g19g5 derivative vector. 14a:BCD2-*ibpA^Pp-^rnpB*T1, position 5, flanked by BpiI restriction sites | This work |
| **Golden gate cloning vectors level 2** | | |
| pAGM4673 | Km^r^, *ori* ColE1^‡^/RK2, Golden gate cloning vector level 2 with canthaxanthin marker, flanked by BpiI restriction sites | (Weber et al., 2011), 48014 |
| pRK99 | Km^r^, Gm^r^, *ori* R6K, Golden gate cloning vector level 2 acceptor backbone adapted from pTn*7*-M with mini-Tn*7* integration sites flanking the cloning site. Contains the canthaxanthin marker flanked by BpiI restriction sites | This work |
| pGG128 | Km^r^, *ori* ColE1^‡^/RK2, pAGM4673 derivative plasmid, empty plasmid (control) | This work |
| pSS126 | Km^r^, *ori* ColE1^‡^/RK2, pAGM4673 derivative plasmid,  14a:BCD2-*phaC2^Rr^*-λT0; 14a:BCD2-*phaA^Rr^*-λT0; 14a:BCD2-*phaB^Rr^*-λT0 | This work |
| pRK182 | Km^r^, *ori* ColE1^‡^/RK2, pAGM4673 derivative plasmid,  14a:BCD2-*phaC2^Rr^*-λT0; 14a:BCD2-*phaA^Rr^*-λT0; 14a:BCD2- *phaB^Rr^*-λT0; λT0-SynPro16-A3283*^Rr^*-λT1; λT0-SynPro16-A2817*^Rr^*-λT1; λT0-SynPro16-A2111*^Rr^*-λT1 | This work |
| pRK216 | Km^r^, *ori* ColE1^‡^/RK2, pAGM4673 derivative plasmid, λT0-SynPro16-*phaC1^Cn^*-*rnpB*T1; λT0-SynPro16-*phaA^Cn^*-*rpoC* term; λT0-SynPro16-*phaB1^Cn^*-T500 | This work |
| pMM85 | Km^r^, *ori* ColE1^‡^/RK2, pAGM4673 derivative plasmid,  14a:BCD2-*phaC1^Cn^-rnpB*T1; 14a:BCD2-*phaA^Cn^-rpoC* term; 14a:BCD2-*phaB^Cn^-*λT1; 14a:BCD2-*phaP1^Cn^*-*rnpB*T1 | This work |
| pMM175 | Km^r^, Gm^r^, *ori* R6K, pRK99 derivative vector; 14a:BCD2- *phaC1^Cn^-rnpB*T1; 14a:BCD2-*phaA^Cn^-rpoC* term; 14a:BCD2-*phaB^Cn^-*λT1 | This work |
| pFB52 | Km^r^, Gm^r^, *ori* R6K, pRK99 derivative vector; 14f:BCD2- *phaC1^Cn^-rnpB*T1; 14a:BCD2-*phaA^Cn^-rpoC* term; 14a:BCD2-*phaB^Cn^-*λT1 | This work |
| pMM106 | Km^r^, *ori* ColE1^‡^/RK2, pAGM4673 derivative plasmid,  14a:BCD2-*phaC5^Pp^*-*rnpB*T1; 14a:BCD2-*phaA3^Pp^*-*rpoC* term; 14a:BCD2-*phaB^Pp^*-λT1; 14a:BCD2-*phaP1^Pp^*-*rnpB*T1 | This work |

Abbreviations: **Gm**, gentamicin; **Sp**, spectinomycin; **Sm**, streptomycin; **Amp**, ampicillin; **Km**, kanamycin; **ColE1^‡^**, ColE1 derivative where the BpiI and BsaI restriction sites were domesticated.

TABLE S2. Primers used in this study

| Primer | Sequence | Part / Purpose | Template (references) |
| --- | --- | --- | --- |
| TS1F | ACCTGCCCGCCGAATTCCTGC | PCR *pha* locus upstream flank | *P. putida* KT2440 genome_PP_5003-PP_5007 locus affected |
| TS1R | GTTTTCCACCACTCATGAGCGTGACCAGTGATAAGGAACA |  |  |
| TS2F | GCTCATGAGTGGTGGAAAACCGC | PCR *pha* locus downstream flank |  |
| TS2R | ATGCAGGATCCTGAATTTGAAACACATGGGGT |  |  |
| FdPHA | TCCCGAGAGATTCTGCCCAT | Genome deletion verification, PCR sequencing primer |  |
| RdPHA | AGCCCGTTCCAGAAGCCGAT |  |  |
| MM388 | TTTGAATTCGACCAAGGTTGAGGGCTACA | PCR *ibpA* locus upstream flank | *P. putida* KT2440 genome_PP_1982 locus affected |
| MM389 | CCTTTTTTTCGTTGCAGCTCGATTTTTGCACGGGGTTTGATGG |  |  |
| MM390 | TCGAGCTGCAACGAAAAAAAGG | PCR *ibpA* locus downstream flank |  |
| MM391 | TTTGGATCCTCACACCACCATGCAGAAGG |  |  |
| MM392 | GGCAAATATCCCGGTGACCT | Genome deletion verification, PCR sequencing primer |  |
| MM393 | GCCGAGCCAATGTCACTG |  |  |
| MM394 | AGTCTTGGAGGCTTGCGTC |  |  |
| RK81 | GGAAGAGCGCCCAATACG | Sequencing of level 0 plasmids | GG/MoClo Level 0 plasmids |
| RK82 | AAAGTGCCACCTGACGTCTA |  |  |
| RK155 | GAACCCTGTGGTTGGCATGCACATAC | Sequencing of level 1 plasmids | GG/MoClo Level 1 plasmids |
| RK156 | CTGGTGGCAGGATATATTGTGGTG |  |  |
| RK157 | GTGGTGTAAACAAATTGACGC | Sequencing of level 2 plasmids | GG/MoClo Level 2 plasmids |
| RK158 | GGATAAACCTTTTCACGCCC |  |  |
| RK247 | TTTGAAGACACcattTAGAACCCCCTCGTACGCTC |  |  |
| SS15 | TTTGAAGACGAaatggcgaccggcaaaggcgcggca | *C. necator phaC1 H16_A1437* | *C. necator H16* genome (Pohlmann et al., 2006) |
| SS16 | TTTGAAGACGAgtcctccatcatgttgcgcacgccggc |  |  |
| SS17 | TTTGAAGACAGggacctgacacgcggcaagatct |  |  |
| SS18 | TTTGAAGACAGaacaccacggcgccttcggtcac |  |  |
| SS19 | TTTGAAGACGTtgttcgagaacgagtacttccagct |  |  |
| SS20 | TTTGAAGACGTcctcgcgcgagccgtagatata |  |  |
| SS21 | TTTGAAGACGAgaggaccatatcgtgccgtggacc |  |  |
| SS22 | TTTGAAGACGAaagctcatgccttggctttgacgtatcg |  |  |
| SS9 | TTTGAAGACGAaatgactgacgttgtcatcgtatccgc | *C. necator phaA H16_A1438* | *C. necator H16* genome (Pohlmann et al., 2006) |
| SS10 | TTTGAAGACGAaagcttatttgcgctcgactgccag |  |  |
| SS11 | TTTGAAGACCTaatgactcagcgcattgcgtatgtgaccg | *C. necator phaB1*  *H16_A1439* | *C. necator H16* genome (Pohlmann et al., 2006) |
| SS12 | TTTGAAGACCTgacaccgtgttgacggtcacgcc |  |  |
| SS13 | TTTGAAGACGAtgtctccgggctatatcgccaccgaca |  |  |
| SS14 | TTTGAAGACGAaagctcagcccatatgcaggccgcc |  |  |
| MM294 | TTTGAAGACGAaatgatcctcaccccggaacaagt | *C. necator phaP1*  *H16_A1381* | *C. necator H16* genome (Pohlmann et al., 2006) |
| MM295 | TTTGAAGACGAaagctcaggcagccgtcgtTttct |  |  |
| RK179 | TTTGAAGACTCaatggccaatcagggcagcgaAaagacccc | *R. rubrum phaC2 Rru_A2413* | *R. rubrum* ATCC 11170 genome (Munk et al., 2011) |
| RK180 | TTTGAAGACTCatacctcgttgttgtcccaggcctcgtcc |  |  |
| RK181 | TTTGAAGACCTgtattcgatttcatcaagcaaacctacc |  |  |
| RK182 | TTTGAAGACCTgaaaacgctatgaccctgatcga |  |  |
| RK183 | TTTGAAGACACtttcGtcatttcgtgggtcaaccccg |  |  |
| RK184 | TTTGAAGACACtcctcgaaggacttggcggcga |  |  |
| RK185 | TTTGAAGACAGaggactatatggtggaaggccccttgg |  |  |
| RK186 | TTTGAAGACAGatcttcgcgggcgctgacgaa |  |  |
| RK187 | TTTGAAGACCTagatcatatcgccccctggcgctcgac |  |  |
| RK188 | TTTGAAGACCTaagcctagccggcgcgcaccttca |  |  |
| RK169 | TTTGAAGACGAaatgaccgatatcgtcattgccggcgc | *R. rubrum phaA Rru_A0274* | *R. rubrum* ATCC 11170 genome (Munk et al., 2011) |
| RK170 | TTTGAAGACGAaagcttagcgctcgacgcagagcg |  |  |
| RK163 | TTTGAAGACGTaatgacgaaagggcgtgtcgctct | *R. rubrum phaB Rru_A0273* | *R. rubrum* ATCC 11170 genome (Munk et al., 2011) |
| RK164 | TTTGAAGACGTaagcttaatacatgtgctggccgcc |  |  |
| RK199 | TTTGAAGACGTaagctcaacgctcgtgaacgtagg |  |  |
| RK255 | TTTGAAGACAGaatgGCCAAGCAACCCGAAACCTTC | *R. rubrum phaP Rru_A3283* | *R. rubrum* ATCC 11170 genome (Munk et al., 2011) |
| RK256 | TTTGAAGACAGaagcTTACTTCTGGGTGGTCGCGG |  |  |
| RK242 | TTTGAAGACGAaatgACGACCCCCCCTAAGAAAAAA | *R. rubrum phaP Rru_A2111* | *R. rubrum* ATCC 11170 genome (Munk et al., 2011) |
| RK243 | TTTGAAGACGAGTCCTCGACGTCAAGCAGGT |  |  |
| RK244 | TTTGAAGACCTGGACCTTCAGAACCTGGAACAG |  |  |
| RK245 | TTTGAAGACCTaagcCTAACCGGCGGCGCCGGC |  |  |
| RK238 | TTTGAAGACTCaatgGTCAATGGCTACGAGGACATCG | *R. rubrum phaP Rru_A2817* | *R. rubrum* ATCC 11170 genome (Munk et al., 2011) |
| RK239 | TTTGAAGACTCaagcTCAGGCGGCCTTGGCGAC |  |  |
| MM286 | TTTGAAGACGTaatgaacatgaacaattcacattct | *P. pseudoalcaligenes phaC5_BN5_4105* | *P. pseudoalcaligenes* CECT5344 genome |
| MM287 | TTTGAAGACGTagAtctccggccagatgcaata |  |  |
| MM288 | TTTGAAGACAGaTctacaagaaggcaagttgcgtc |  |  |
| MM289 | TTTGAAGACAGaagctcattgcttcacgtagcgc |  |  |
| MM304 | CATCACCTGGGAGCAGATCAT |  |  |
| MM280 | TTTGAAGACAGaatgatcgacgtcgttatcgtcgc | *P. pseudoalcaligenes phaA3_BN5_4104* | *P. pseudoalcaligenes* CECT5344 genome |
| MM281 | TTTGAAGACAGcTggggcgatacccatgatcg |  |  |
| MM282 | TTTGAAGACCAccAgtctcggccagccgccac |  |  |
| MM283 | TTTGAAGACCAaagctcagcgttcaatggccagc |  |  |
| MM284 | TTTGAAGACGAaatggccacttcgagtaatccgac | *P. pseudoalcaligenes phaB_BN5_4103* | *P. pseudoalcaligenes* CECT5344 genome |
| MM285 | TTTGAAGACGAaagctcactgcatattcatgccgc |  |  |
| MM290 | TTTGAAGACAGaatgtctttttttgattcggaaaaactg | *P. pseudoalcaligenes phaP_BN5_4096* | *P. pseudoalcaligenes* CECT5344 genome |
| MM291 | TTTGAAGACAGacGagatcgtacacctcgcgat |  |  |
| MM292 | TTTGAAGACTGtCgtctccagcacgcaggcgga |  |  |
| MM293 | TTTGAAGACTGaagcttacttgcgcccgcccgc |  |  |
| MM397 | TTTGAAGACTGaatgaccatgactactgctttctctct | *P. putida ibpA*_ PP_1982 | *P. putida* KT2440 genome |
| MM398 | TTTGAAGACTGaagctcagttcagcgctggttttt |  |  |

Underlined nucleotides represent BbsI recognition sites for Golden gate parts, otherwise EcoRI, BamHI, NotI or NcoI sites; lower case letters represent nucleotides corresponding to Golden gate fusion sites.

**Genome sequencing of *pha* cluster-deleted PP05_01 strain**

For the *pha* cluster deletion (PP05_01 strain), the pEMG knockout system was used, with some modifications (Martínez-García and de Lorenzo, 2011b). Briefly, two pairs of primers (TS1F-TS1R, TS2F-TS2R, Table S2) were designed to amplify the flanking fragments of the *pha* cluster (affected locus PP_5003-PP_5008). An overlap PCR was carried out giving a product of 1 Kb (TS1-TS2 homology regions) that was cloned into pEMG plasmid using EcoRI and BamHI restriction enzymes. The constructing vector pEMG incorporates two I-Sce-I sites (5´-TAGGGATAACAGGGTAAT-3´) flanking a lacZa poly-linker. This vector functions in concert with the I-Sce-I producing pSW(I-SceI) plasmid that allows the accumulative edition of the genome of *P. putida* (Martínez-García and de Lorenzo, 2011b). *P. putida* transconjugants of the first recombination event were transformed with pSW-I via electroporation. The selection plates were LB + 500 μg/mL Amp and 15 mM 3-methylbenzoate (3MB) and incubated at 30 ºC for 16 h. The 3MB was used to induce the I-Sce-I system. Single colonies were picked on LB + 500 μg/mL Amp and LB + Km to screen for kanamycin sensitive clones. Kanamycin sensitive clones were checked for the knockout phenotypes, using external primers to the homology region (FdPHA and RdPHA, Table S2). To confirm the integrity of the homology region, DNA sequencing of the PCR product was also performed. Under non-selective cultivation *Pseudomonas* loses the pSW-I plasmid quite fast. For this purpose, several single colonies were checked for 500 μg/mL Amp sensitivity to verify pSW-I plasmid loss. Genome sequencing reads for the PP05_01 strain was deposited in the National Center for Biotechnology Information Sequence Reads Archive under the accession number SRX3133083 (2017) (<http://www.ncbi.nlm.nih.gov/sra>). This strain has been deposited at the Spanish type culture collection with the code number CECT 30020 (Mato et al., 2020).

The deletion of the entire 6373 bp *pha* locus was confirmed by PCR and further validated by genomic sequencing of PP05_01 along with the wild type parental KT2440 strain used in our laboratory (Figure S1). Common to both Belda et al., and our sequencing, 30 polymorphisms were observed in noncoding regions and one further single base insertion was found in the coding region of the pseudogene PP_0253 (Belda et al., 2016). Additional polymorphisms were found only in our laboratory’s wild type and PP05_01 strains, with a single base insertion in PP_0278, which encodes a hypothetical protein, and a synonymous substitution in PP_4866 (*braD*, high-affinity branched-chain amino acid ABC transporter permease) (Table S3). These variations from Belda et al sequence are in keeping with the tendency of laboratory strains to evolve over time away from a founder strain (Barrick et al., 2009). When comparing the sequencing between our wild type KT2440 and PP05_01 strains, the expected deletion of the *pha* locus was verified in the PP05_01 strain. Moreover, an additional single base deletion appeared in PP05_01 that was not seen in KT2440. This mutation was in PP_3691, which encodes a DNA helicase-related protein and results in its premature truncation. PP_3691 encodes a large 245 kDa protein with the deletion mutation near its midpoint. It is not known if this mutation affects the function of this protein. The PP_3691 gene is distal to the *pha* locus (PP_5003-PP_5008) and it is not known how this new mutation came about, but may have arisen during the conjugation and selection steps carried out during *pha* locus deletion.


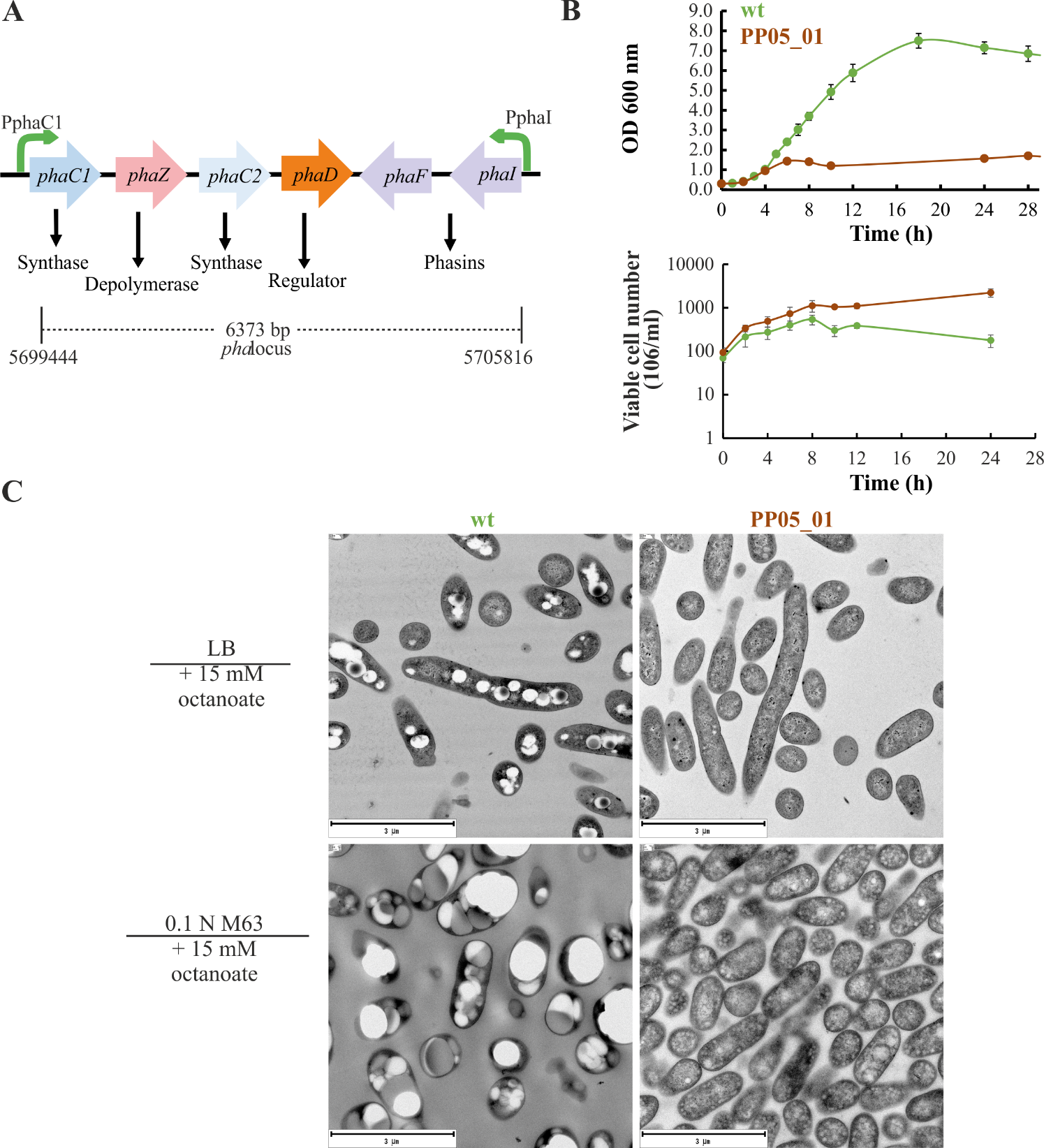


**FIGURE S1. Deletion of *pha* gene locus in *P. putida*. A.** The *pha* gene locus in *P. putida* contains two convergently oriented operons. Left operon: *phaC1, phaZ, phaC2 and phaD*. Right operon: *phaF* and *phaI*. The promoters for *phaC1* and *phaI* are indicated. Genome coordinates indicate the boundary of the Δ*pha* deletion (PP05_01 strain) and are based on the *P. putida* KT2440 complete genome GenBank version AE015451.2. **B.** Growth characteristics of *P. putida* Δ*pha*, PP05_01. Growth as determined by growth curve (OD 600 nm) and colony forming units (CFU, cell viable cell number) of wild type (wt, green color) and PP05_01 (brown color) strains in 0.1 N M63 minimal media with 15 mM octanoate (see Materials and methods). Data is the mean value from at least three replicates and were obtained from (Manoli et al., 2022). **C.** Transmission electron micrographs of wt and PP05_01 strains grown 24 h in LB or 0.1 N M63 minimal media each supplemented with 15 mM octanoate. Scale bars represent 3 µm.

**TABLE S3.** Mutations found resequencing *P. putida* KT2440

| **START** | **END** | **TYPE** | **Mutation** | **IN Coding region?** | ***P. putida* KT2440** | ***P. putida* KT2440 Δ*pha, PP05_01*** | **ORF** | **Description** |
| --- | --- | --- | --- | --- | --- | --- | --- | --- |
| 278743 | 278743 | I | A | NO | YES | YES |  |  |
| **307866** | **307866** | **I** | **C** | **YES** | **YES** | **YES** | ***PP_0253*** | ***pck*, phosphoenolpyruvate carboxykinase pseudogene** |
| **336125** | **336125** | **I** | **T** | **YES** | **YES** | **YES** | ***PP_0278*** | **hypothetical protein** |
| 353067 | 353067 | S | A>G | NO | YES | YES |  |  |
| 499203 | 499203 | S | A>G | NO | YES | YES |  |  |
| 1070247 | 1070247 | I | GA | NO | YES | YES |  |  |
| 1126646 | 1126646 | I | C | NO | YES | YES |  |  |
| 1419549 | 1419549 | I | C | NO | YES | YES |  |  |
| 1499480 | 1499480 | S | C>A | NO | YES | YES |  |  |
| 1499481 | 1499481 | S | A>C | NO | YES | YES |  |  |
| 1499498 | 1499498 | S | T>G | NO | YES | YES |  |  |
| 1499498 | 1499498 | I | C | NO | YES | YES |  |  |
| 1499507 | 1499507 | I | C | NO | YES | YES |  |  |
| 1932639 | 1932639 | I | C | NO | YES | YES |  |  |
| 3845704 | 3845704 | I | G | NO | YES | YES |  |  |
| 3951159 | 3951159 | S | T>C | NO | YES | YES |  |  |
| 3951161 | 3951161 | S | A>T | NO | YES | YES |  |  |
| **4203482** | **4203482** | **D** | **G>** | **YES** | **NO** | **YES** | ***PP_3691*** | **DNA helicase-related protein** |
| 4586030 | 4586030 | S | C>T | NO | YES | YES |  |  |
| 4586031 | 4586031 | S | T>C | NO | YES | YES |  |  |
| 4586034 | 4586034 | I | G | NO | YES | YES |  |  |
| 4586057 | 4586057 | I | C | NO | YES | YES |  |  |
| 4740805 | 4740805 | D | T> | NO | YES | YES |  |  |
| 4740816 | 4740816 | S | T>G | NO | YES | YES |  |  |
| 4740819 | 4740819 | S | T>C | NO | YES | YES |  |  |
| 4740820 | 4740820 | S | G>T | NO | YES | YES |  |  |
| 4980586 | 4980586 | I | GGC | NO | YES | YES |  |  |
| **5532443** | **5532443** | **S** | **G>A** | **YES** | **YES** | **YES** | ***PP_4866*** | ***braD*, high-affinity branched-chain amino acid ABC transporter permease. Encodes synonymous mutation.** |
| 5555609 | 5555609 | I | GCC | NO | YES | YES |  |  |
| 5674751 | 5674751 | I | G | NO | YES | YES |  |  |
| 5674753 | 5674753 | S | G>C | NO | YES | YES |  |  |
| 5681416 | 5681416 | I | CGGG | NO | YES | YES |  |  |
| **5699443** | **5705817** | **D** |  | **YES** | **NO** | **YES** | ***phaC1-phaI* locus deletion** | ***Δpha*** |
| 6013885 | 6013885 | S | T>C | NO | YES | YES |  |  |

Results from resequencing our laboratory stocks of *P. putida* KT2440 and Δ*pha,* PP05_01 strains*.* Mutations in coding regions are highlighted in bold. Abbreviations: **S**, substitution; **I**, insertion; **D**, deletion. Start and end coordinates are from *P. putida* KT2440 complete genome GenBank version AE015451.2.


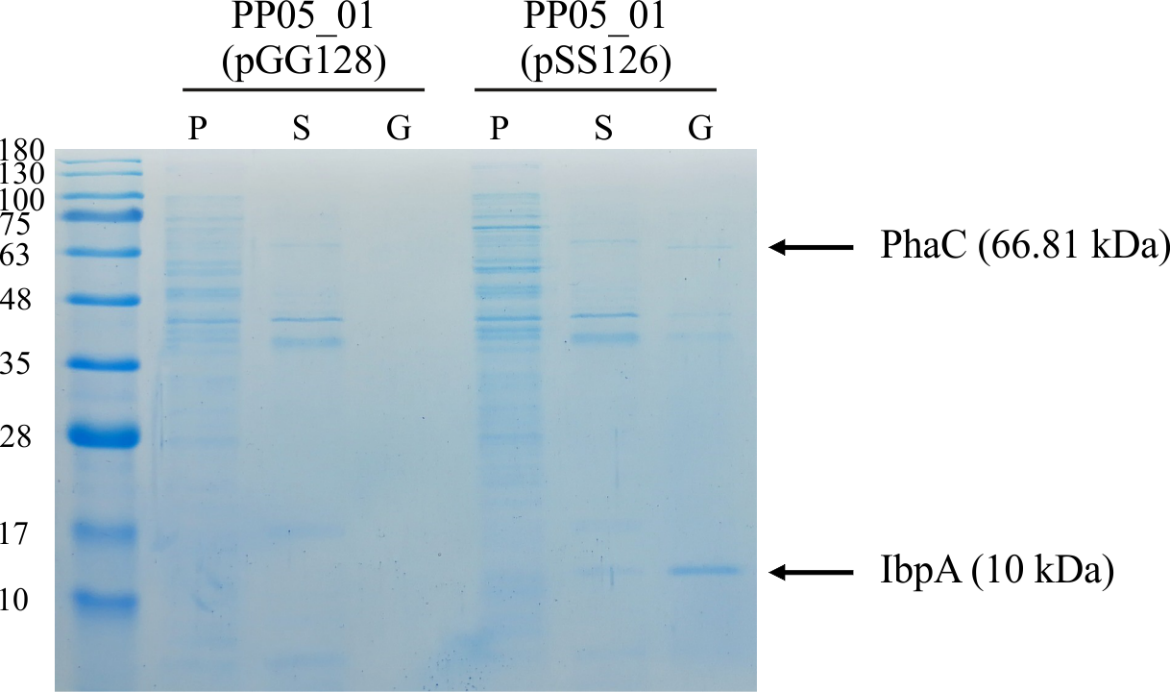


**FIGURE S2. SDS-PAGE analysis of PHA granule fractions.** In PP05_01 derivative strains harboring pGG128 (empty plasmid) and pSS126 (p*haCAB*^Rr^), a band corresponding to the size of PhaC2^Rr^ (66.81 kDa) can be detected in granule fractions from strains containing *phaC2* (harboring pSS126) and a lower fraction of IbpA (10 kDa) shown with black arrows. PhaA and PhaB were not detected at any fraction. P: pellet, S: supernatant, G: granule fractions. The MW of the corresponding GAPs are indicated. BlueStar Prestained protein marker (Nippon Genetics) was used as a molecular weight marker using Tris-Glycine 4-20% conditions.

**
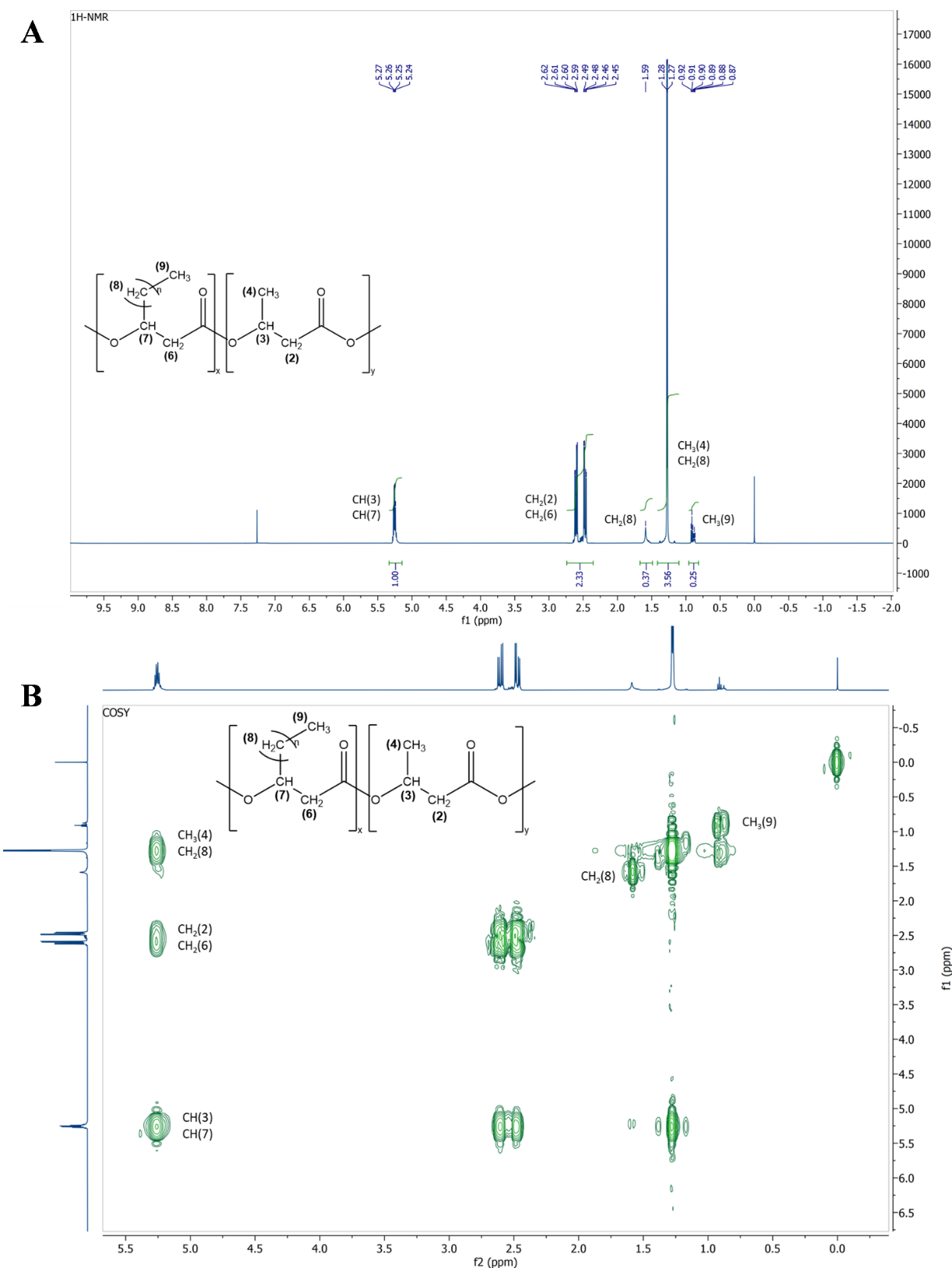
**

**FIGURE S3. NMR spectroscopy for structural confirmation of PHA.** ^1^H-NMR (A) and COSY (B) spectra of the obtained pure PHA. CDCl_3_ and TMS were used as a solvent and an internal standard, respectively. The chemical shifts are in ppm.

***SUPPLEMENTAL REFERENCES***

Barrick, J. E., Yu, D. S., Yoon, S. H., Jeong, H., Oh, T. K., Schneider, D., et al. (2009). Genome evolution and adaptation in a long-term experiment with Escherichia coli. *Nature* 461, 1243–1247. doi: 10.1038/nature08480.

Belda, E., van Heck, R. G. A., José Lopez-Sanchez, M., Cruveiller, S., Barbe, V., Fraser, C., et al. (2016). The revisited genome of Pseudomonas putida KT2440 enlightens its value as a robust metabolic chassis. *Environmental Microbiology* 18. doi: 10.1111/1462-2920.13230.

Blanco, F. G., Machatschek, R., Keller, M., Hernández-Arriaga, A. M., Godoy, M. S., Tarazona, N. A., et al. (2023). Nature-inspired material binding peptides with versatile polyester affinities and binding strengths. *International Journal of Biological Macromolecules* 253, 126760. doi: 10.1016/j.ijbiomac.2023.126760.

Blázquez, B., León, D. S., Torres-Bacete, J., Gómez-Luengo, Á., Kniewel, R., Martínez, I., et al. (2023). Golden Standard: a complete standard, portable, and interoperative MoClo tool for model and non-model proteobacteria. *Nucleic Acids Research*, gkad758. doi: 10.1093/nar/gkad758.

Choi, K.-H., Gaynor, J. B., White, K. G., Lopez, C., Bosio, C. M., Karkhoff-Schweizer, R. R., et al. (2005). A Tn7-based broad-range bacterial cloning and expression system. *Nat Methods* 2, 443–448. doi: 10.1038/nmeth765.

Manoli, M.-T., Nogales, J., and Prieto, A. (2022). Synthetic Control of Metabolic States in Pseudomonas putida by Tuning Polyhydroxyalkanoate Cycle. *mBio* 13, e01794-21. doi: 10.1128/mbio.01794-21.

Martínez-García, E., and de Lorenzo, V. (2011a). Engineering multiple genomic deletions in Gram-negative bacteria: analysis of the multi-resistant antibiotic profile of *Pseudomonas putida* KT2440. *Environ. Microbiol.* 13, 2702–2716. doi: 10.1111/j.1462-2920.2011.02538.x.

Martínez-García, E., and de Lorenzo, V. (2011b). Engineering multiple genomic deletions in Gram-negative bacteria: Analysis of the multi-resistant antibiotic profile of Pseudomonas putida KT2440. *Environmental Microbiology*. doi: 10.1111/j.1462-2920.2011.02538.x.

Mato, A., Blanco, F. G., Maestro, B., Sanz, J. M., Pérez-Gil, J., and Prieto, M. A. (2020). Dissecting the Polyhydroxyalkanoate-Binding Domain of the PhaF Phasin: Rational Design of a Minimized Affinity Tag. *Appl Environ Microbiol* 86, e00570-20. doi: 10.1128/AEM.00570-20.

Munk, A. C., Copeland, A., Lucas, S., Lapidus, A., Del Rio, T. G., Barry, K., et al. (2011). Complete genome sequence of *Rhodospirillum rubrum* type strain (S1T). *Stand Genomic Sci* 4, 293–302. doi: 10.4056/sigs.1804360.

Pohlmann, A., Fricke, W. F., Reinecke, F., Kusian, B., Liesegang, H., Cramm, R., et al. (2006). Genome sequence of the bioplastic-producing “Knallgas” bacterium *Ralstonia eutropha* H16. *Nat. Biotechnol.* 24, 1257–1262. doi: 10.1038/nbt1244.

Weber, E., Engler, C., Gruetzner, R., Werner, S., and Marillonnet, S. (2011). A Modular Cloning System for Standardized Assembly of Multigene Constructs. *PLOS ONE* 6, e16765. doi: 10.1371/journal.pone.0016765.

Zobel, S., Benedetti, I., Eisenbach, L., De Lorenzo, V., Wierckx, N., and Blank, L. M. (2015). Tn7-Based Device for Calibrated Heterologous Gene Expression in Pseudomonas putida. *ACS Synthetic Biology* 4, 1341–1351. doi: 10.1021/acssynbio.5b00058.
